# Supplementary material for: Transcriptomic mapping of the 5-HT receptor landscape
Source: Patterns (N Y). 2024 Aug 29;5(10):101048. doi: 10.1016/j.patter.2024.101048 (PMC11574285; doi:10.1016/j.patter.2024.101048)
Supplement: Document S1. Figures S1–S10 and Tables S1 and S3 [file mmc1.pdf]

Patterns, Volume *5*

Supplemental information

Transcriptomic mapping of the 5-HT receptor landscape

Roberto De Filippo and Dietmar Schmitz

Transcriptomic Mapping of the 5-HT Receptor Landscape

Roberto De Filippo[1] and Dietmar Schmitz[1][2][3][4][5]

[1] Charité Universitätsmedizin Berlin, corporate member of Freie Universität Berlin, Humboldt-Universität zu Berlin, and Berlin Institute of Health; Neuroscience Research Center, 10117 Berlin, Germany.
[2] German Center for Neurodegenerative Diseases (DZNE) Berlin, 10117 Berlin, Germany.
[3] Charité-Universitätsmedizin Berlin, corporate member of Freie Universität Berlin, Humboldt-Universität Berlin, and Berlin Institute of Health, Einstein Center for Neuroscience, 10117 Berlin, Germany.
[4] Charité-Universitätsmedizin Berlin, corporate member of Freie Universität Berlin, Humboldt-Universität Berlin, and Berlin Institute of Health, NeuroCure Cluster of Excellence, 10117 Berlin, Germany.
[5] Humboldt-Universität zu Berlin, Bernstein Center for Computational Neuroscience, Philippstr. 13, 10115 Berlin, Germany.

* Corresponding author and lead contact: roberto.de-filippo@bccn-berlin.de

Supplemental Information

Supplementary Figures

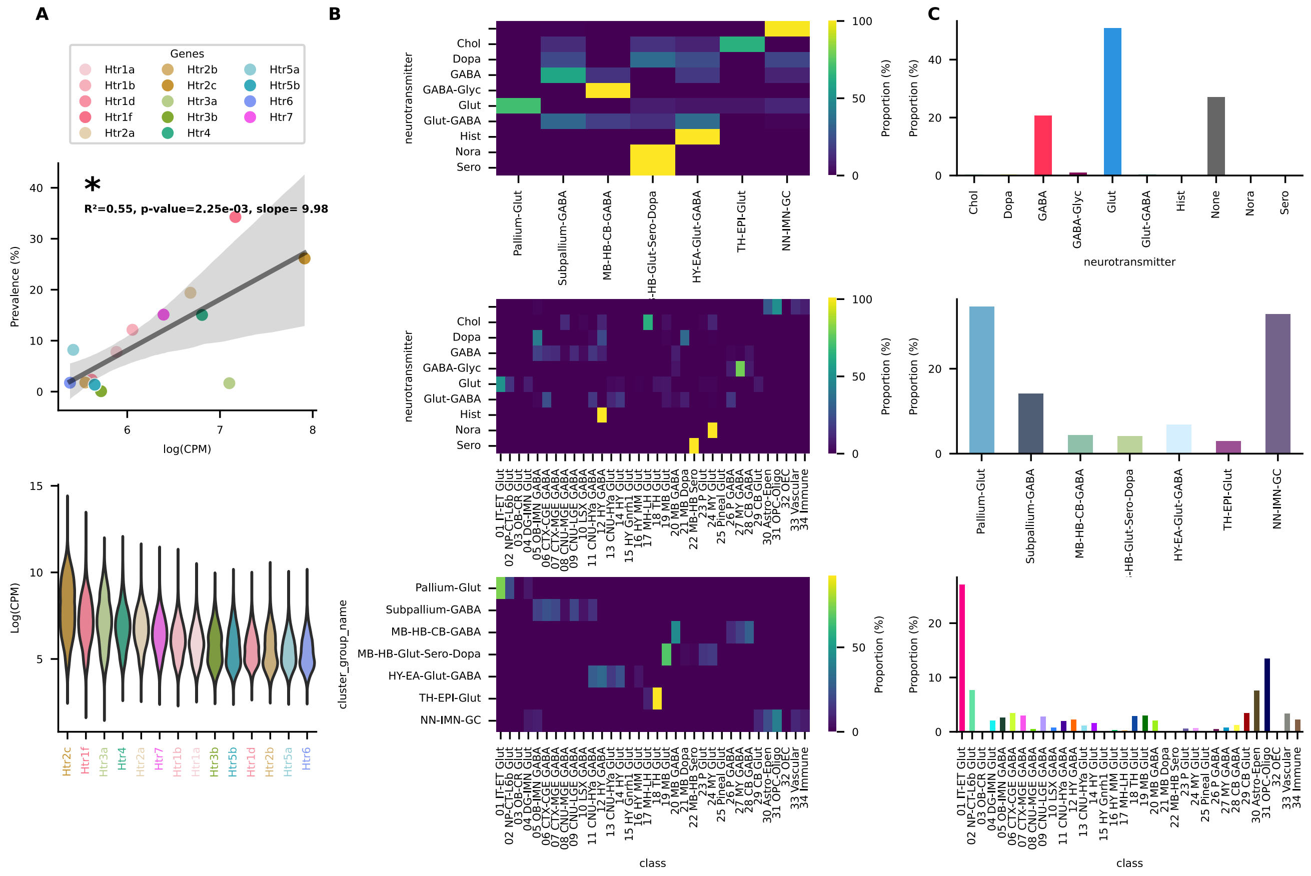

Figure S1. Htrs transcription and hierarchical clustering visualization.

(A) Top: Linear regression model fit between prevalence (y axis) and amount of intracellular expression (x axis) for each Htrs. Bottom: violin plots showing distribution of intracellular amount of expression for each Htrs. (B) Heatmaps showing relationship between neurotransmitter, class and neighborhoods. (C) Bar plots representing the size of each group when cells are categorized by neurotransmitter (top), neighborhood (middle) and class (botttom).

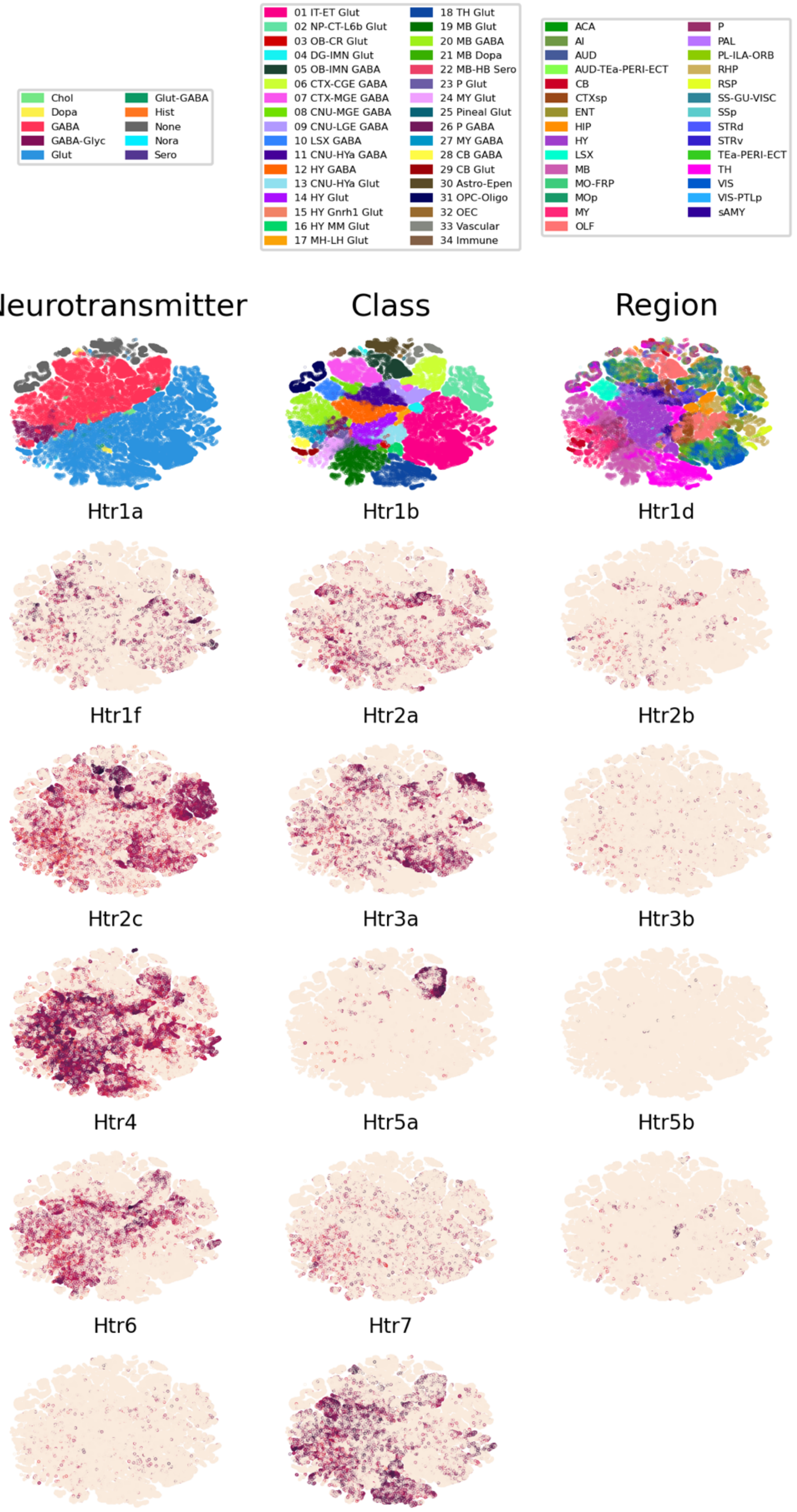

27

28 **Figure S2. UMAP distribution of each Htr.**

29 UMAP-associated neurotransmitter, class and dissection region metadata (first row) and

30 UMAP distribution of each Htr.

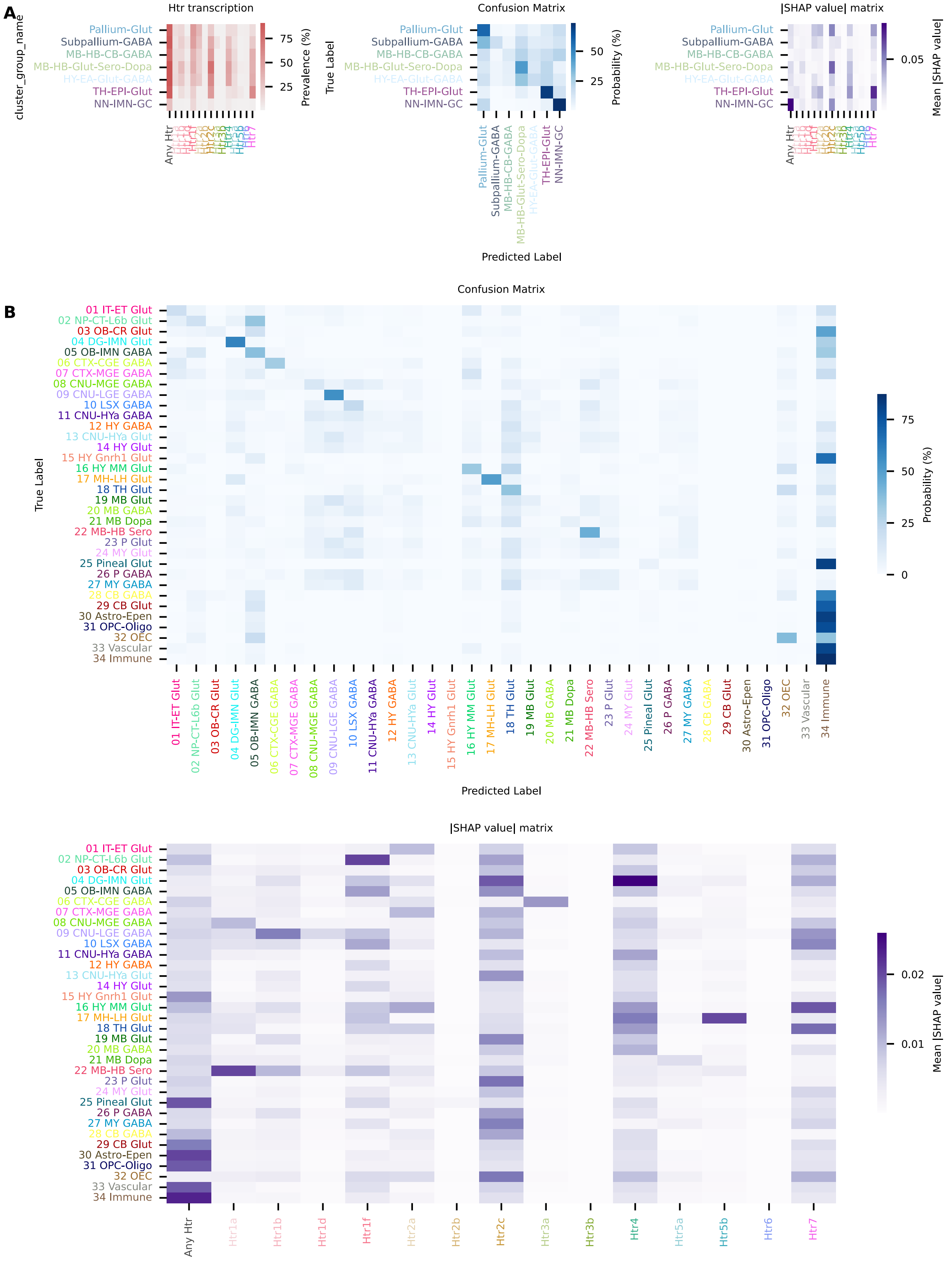

Figure S3. Htrs features across neighborhoods and classes.

(A) Htrs prevalence across neighborhoods (left). Confusion matrix related to the decoding of neighboroods classification from Htrs transcription (middle). SHAP values matrix associated to the neighborhood decoding (right). (B) Confusion matrix related to the decoding of classes

classification from Htrs transcription (left). SHAP values matrix associated to the class decoding (right).

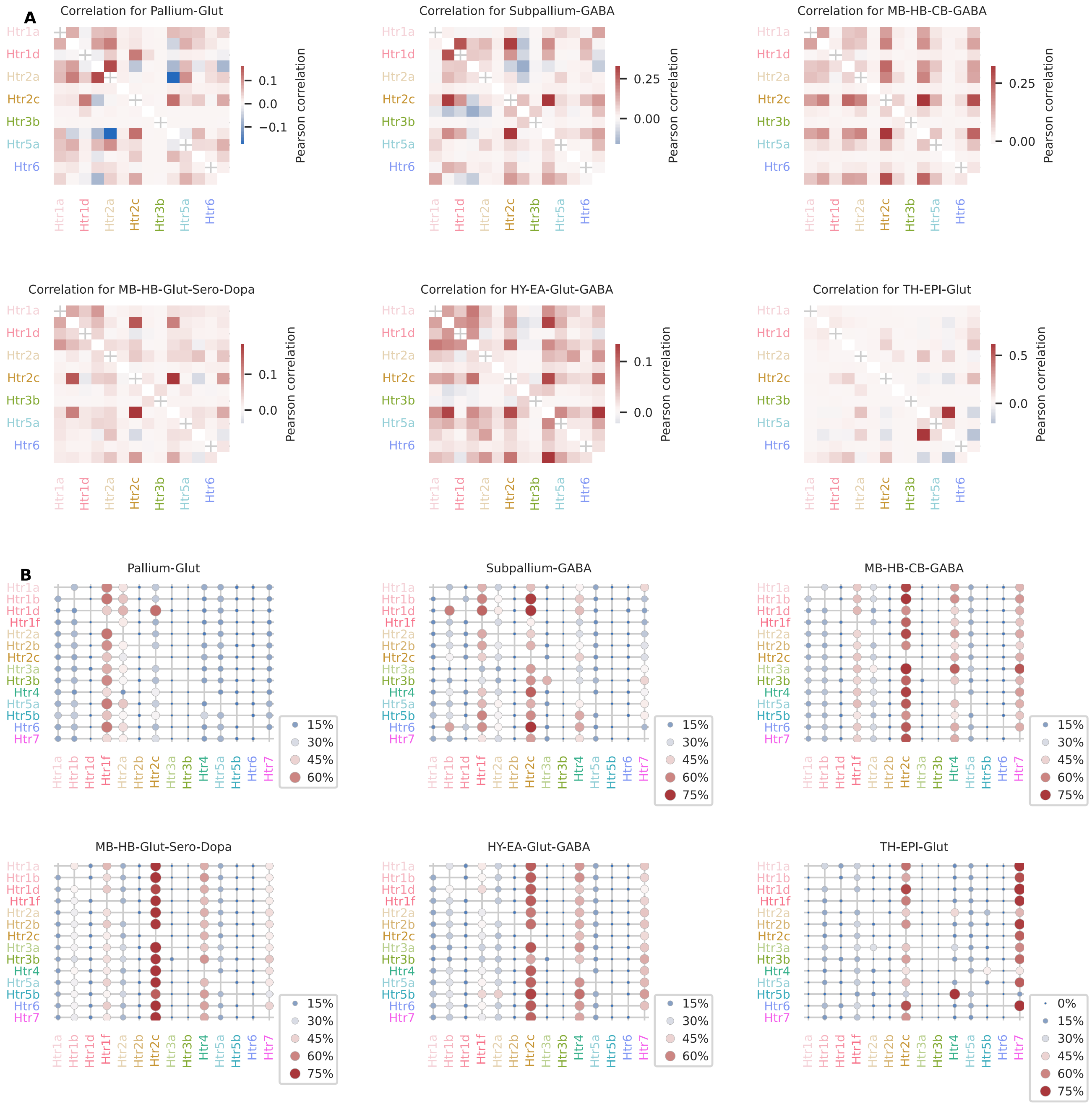

Figure S4. Correlation and co-localization patterns across neighborhoods.

(A) Correlation matrices between pairs of Htrs in each neighborhood. (B) co-localization matrices of Htrs transcription in each neighborhood.

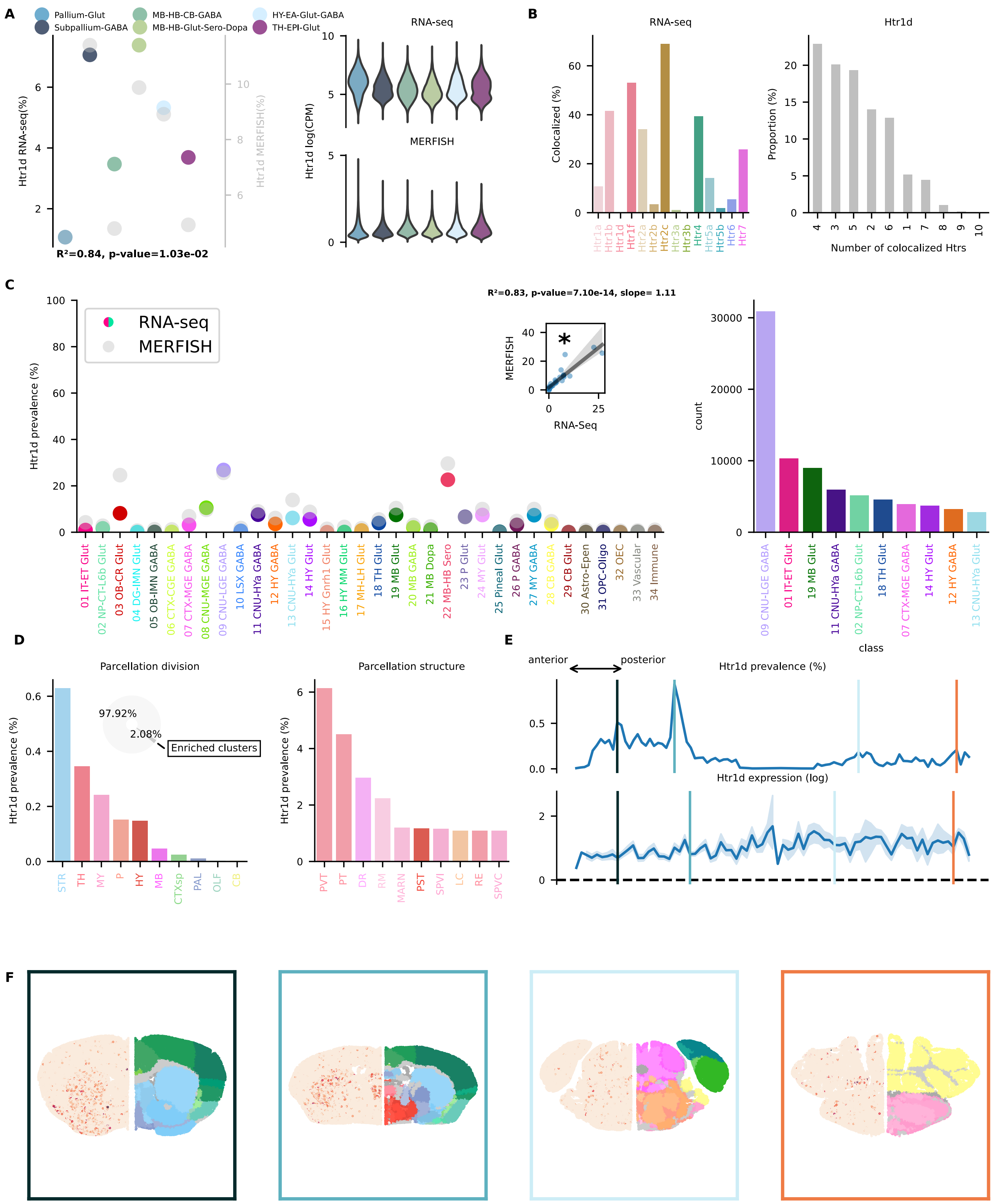

Figure S5. Htr1d transcription.

(A) On the left, Htr1d prevalence across neighborhoods with squared Pearson correlation coefficient (R^2) between scRNA-seq and MERFISH dataset. On the right, amount of Htr1d RNA detected using scRNA-seq (top) and MERFISH (bottom). (B) Amount of co-localization with each Htrs by cells expressing Htr1d RNA (left). Number of Htrs RNA detected in cells expressing Htr1d RNA (right). (C) Prevalence of Htr1d RNA across all classes of cells in

scRNA-seq and MERFISH dataset. Inset represents the linear regression between the two datasets. On the right, absolute number of cells expressing Htr1d RNA by class ranked in descending order (top ten). (D) Prevalence of Htr1d RNA across divisions (left) and structures (right). Inset represents the proportion of cells expressing Htr1d RNA that belongs to enriched clusters. (E) Top: Prevalence of cells from enriched clusters across the antero-posterior axis, identified in the scscRNA-seq dataset and cross-referenced in the MERFISH dataset. Bottom: average amount of RNA expression found in enriched clusters cross-referenced in the MERFISH dataset. (F) Expression of Htr1d RNA detected by MERFISH in 4 representative slices. Border color represents the position on the antero-posterior axis.

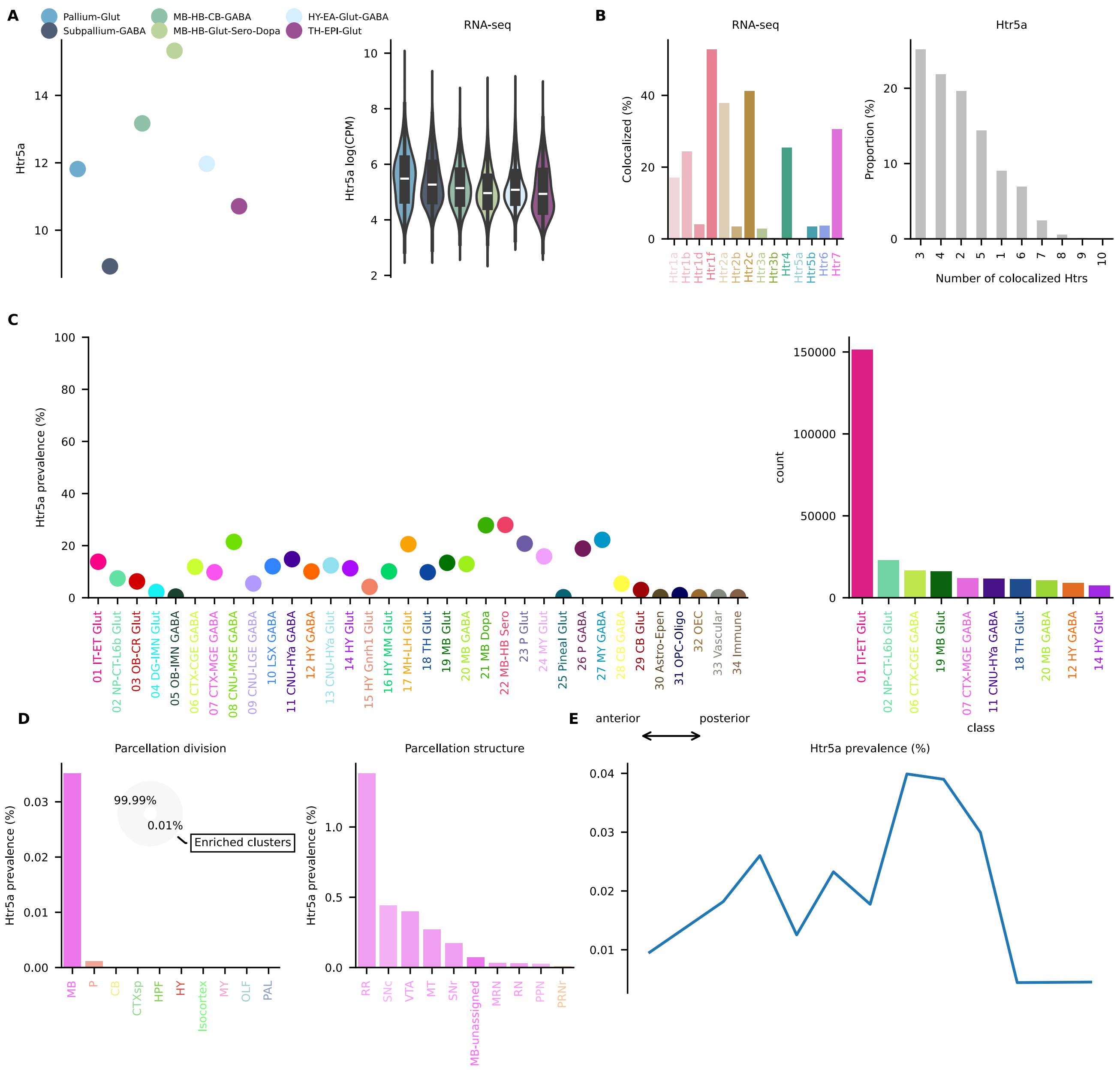

Supplementary Figure S6. Htr5a transcription.

(A) On the left, Htr5a prevalence across neighborhoods with squared Pearson correlation coefficient (R^2) between scRNA-seq and MERFISH dataset. On the right, amount of Htr5a RNA detected using scRNA-seq (top) and MERFISH (bottom). (B) Amount of co-localization with each Htrs by cells expressing Htr5a RNA (left). Number of Htrs RNA detected in cells expressing Htr5a RNA (right). (C) Prevalence of Htr5a RNA across all classes of cells in scRNA-seq and MERFISH dataset. Inset represents the linear regression between the two datasets. On the right, absolute number of cells expressing Htr5a RNA by class ranked in descending order (top ten). (D) Prevalence of Htr5a RNA across divisions (left) and structures (right). Inset represents the proportion of cells expressing Htr5a RNA that belongs to enriched clusters. (E)

Prevalence of cells from enriched clusters across the antero-posterior axis, identified in the scscRNA-seq dataset and cross-referenced in the MERFISH dataset.

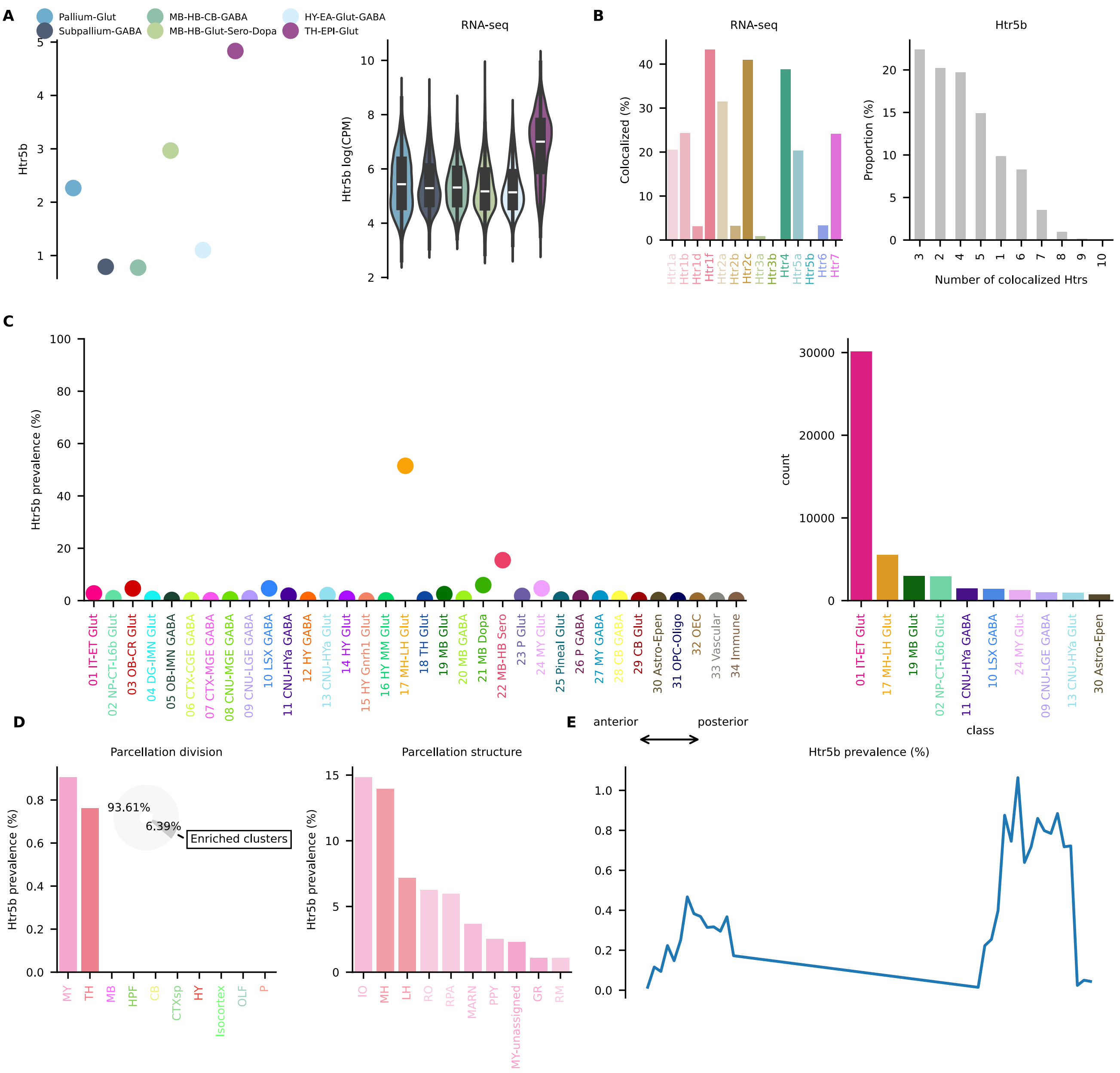

Figure S7. Htr5b transcription.

(A) On the left, Htr5b prevalence across neighborhoods with squared Pearson correlation coefficient (R^2) between scRNA-seq and MERFISH dataset. On the right, amount of Htr5b RNA detected using scRNA-seq (top) and MERFISH (bottom). (B) Amount of co-localization with each Htrs by cells expressing Htr5b RNA (left). Number of Htrs RNA detected in cells expressing Htr5b RNA (right). (C) Prevalence of Htr5b RNA across all classes of cells in scRNA-seq and MERFISH dataset. Inset represents the linear regression between the two datasets. On the right, absolute number of cells expressing Htr5b RNA by class ranked in descending order (top ten). (D) Prevalence of Htr5b RNA across divisions (left) and structures (right). Inset represents the proportion of cells expressing Htr5b RNA that belongs to enriched

clusters. (E) Prevalence of cells from enriched clusters across the antero-posterior axis, identified in the scscRNA-seq dataset and cross-referenced in the MERFISH dataset.

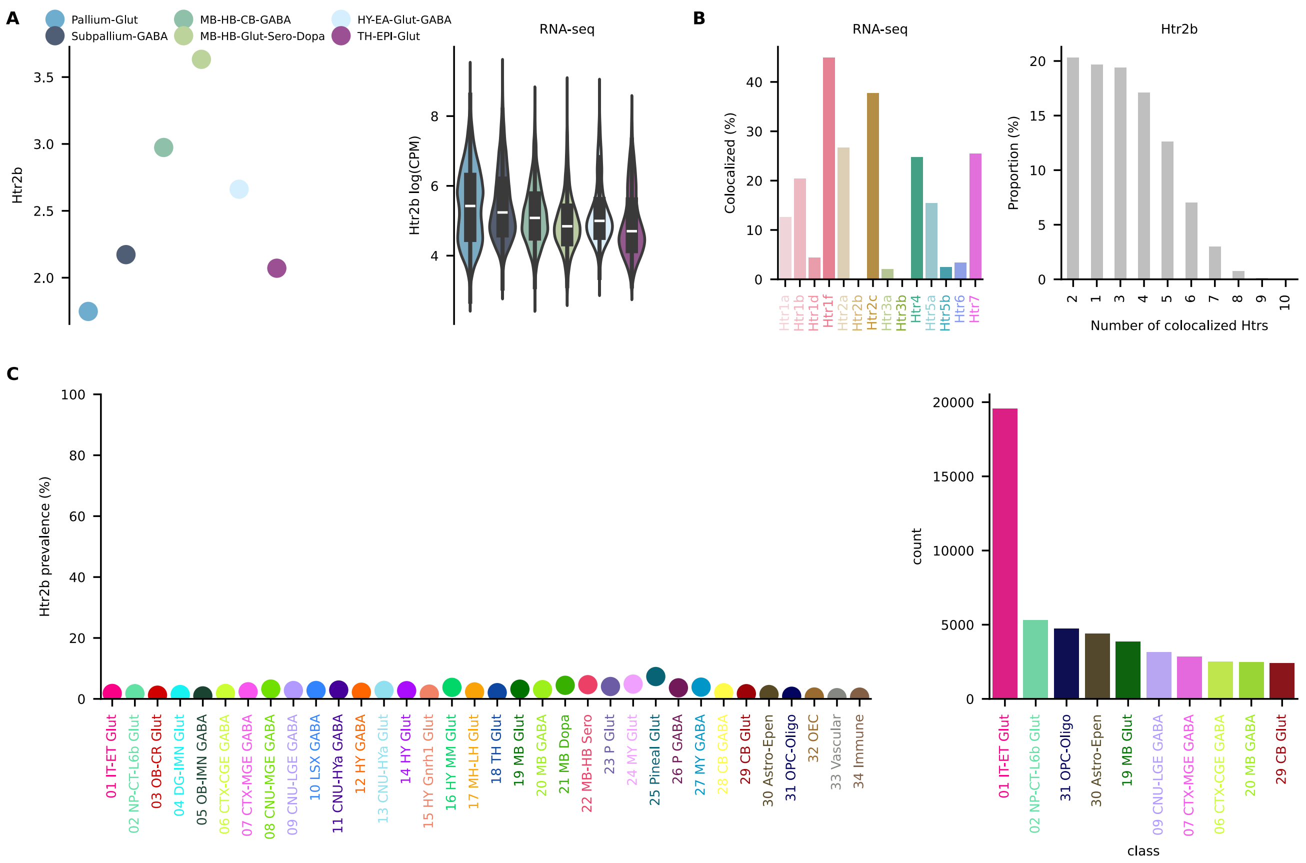

Supplementary Figure S8. Htr2b transcription.

(A) On the left, Htr2b prevalence across neighborhoods with squared Pearson correlation coefficient (R^2) between scRNA-seq and MERFISH dataset. On the right, amount of Htr2b RNA detected using scRNA-seq (top) and MERFISH (bottom). (B) Amount of co-localization with each Htrs by cells expressing Htr2b RNA (left). Number of Htrs RNA detected in cells expressing Htr2b RNA (right). (C) Prevalence of Htr2b RNA across all classes of cells in scRNA-seq and MERFISH dataset. Inset represents the linear regression between the two datasets. On the right, absolute number of cells expressing Htr2b RNA by class ranked in descending order (top ten).

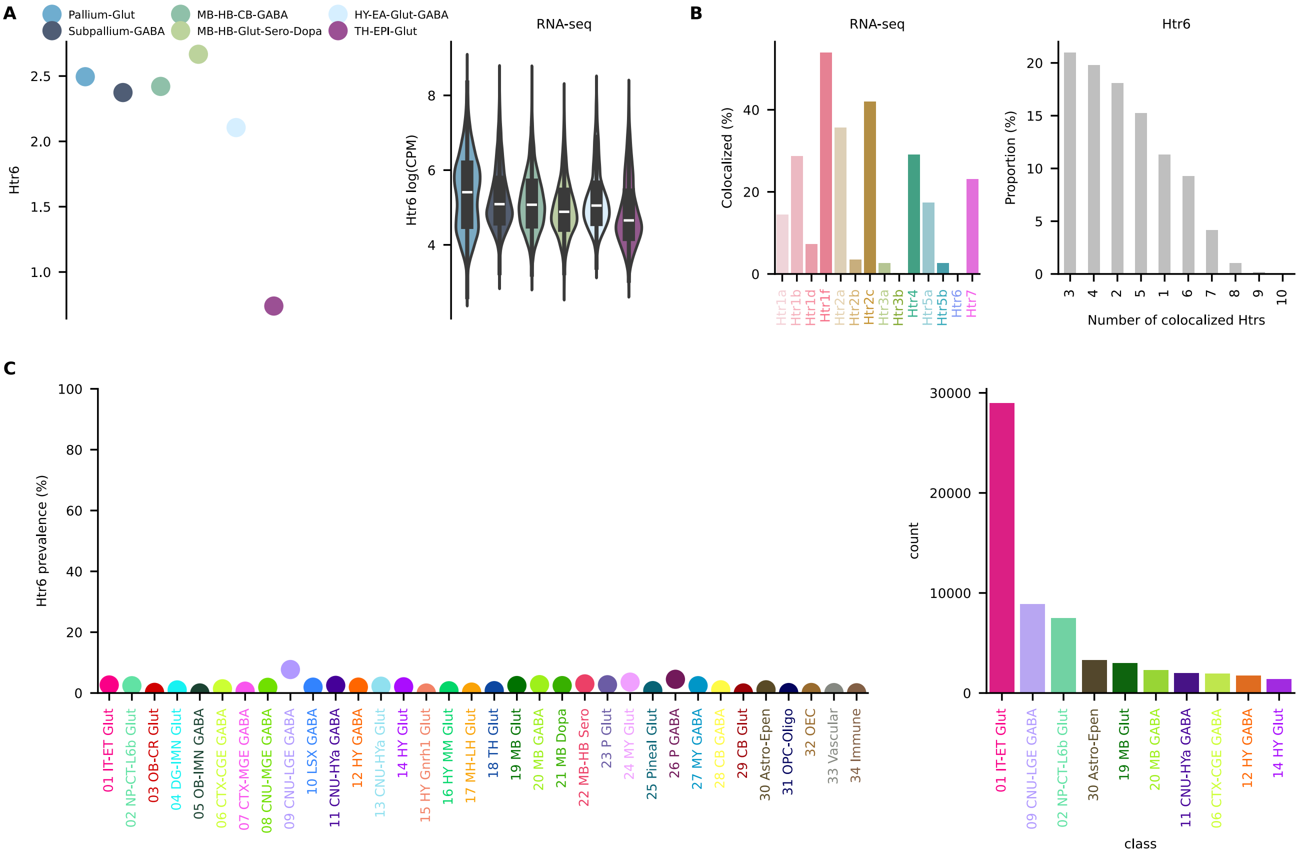

Figure S9. Htr6 transcription.

(A) On the left, Htr6 prevalence across neighborhoods with squared Pearson correlation coefficient (R^2) between scRNA-seq and MERFISH dataset. On the right, amount of Htr6 RNA detected using scRNA-seq (top) and MERFISH (bottom). (B) Amount of co-localization with each Htrs by cells expressing Htr6 RNA (left). Number of Htrs RNA detected in cells expressing Htr6 RNA (right). (C) Prevalence of Htr6 RNA across all classes of cells in scRNA-seq and MERFISH dataset. Inset represents the linear regression between the two datasets. On the right, absolute number of cells expressing Htr6 RNA by class ranked in descending order (top ten).

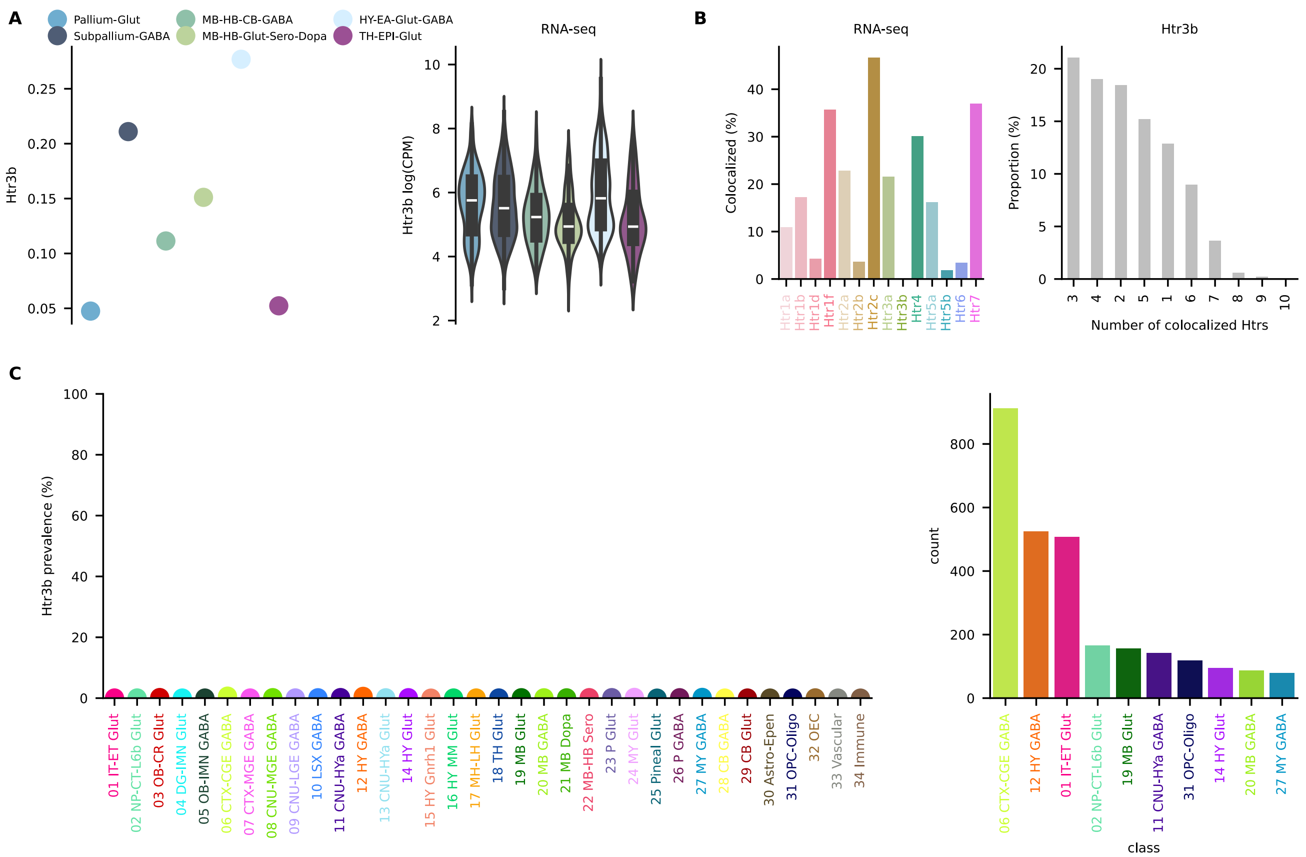

Figure S10. Htr3b transcription.

(A) On the left, Htr3b prevalence across neighborhoods with squared Pearson correlation coefficient (R^2) between scRNA-seq and MERFISH dataset. On the right, amount of Htr3b RNA detected using scRNA-seq (top) and MERFISH (bottom). (B) Amount of co-localization with each Htrs by cells expressing Htr3b RNA (left). Number of Htrs RNA detected in cells expressing Htr3b RNA (right). (C) Prevalence of Htr3b RNA across all classes of cells in scRNA-seq and MERFISH dataset. Inset represents the linear regression between the two datasets. On the right, absolute number of cells expressing Htr3b RNA by class ranked in descending order (top ten).

name	description
**HY-EA-Glut-GABA**	Glutamatergic and GABAergic neurons in hypothalamus and extended amygdala, also including all non-pallium glutamatergic neurons in cerebral nuclei (1404 clusters)
**MB-HB-CB-GABA**	GABAergic neurons in midbrain, hindbrain, and cerebellum (1040 clusters)
**MB-HB-Glut-Sero-Dopa**	Glutamatergic, serotonergic and dopaminergic neurons in midbrain and hindbrain (1431 clusters)
**NN-IMN-GC**	Non-neuronal cells, immature neurons, granule cells in dentate gyrus, olfactory bulb and cerebellum, and neurons in pineal gland (248 clusters)
**Pallium-Glut**	Glutamatergic neurons from cerebral cortex (derived from the developmental pallium), including isocortex, hippocampal formation, olfactory areas, and cortical subplate (517 clusters)
**Subpallium-GABA**	Telencephalic GABAergic neurons from cerebral cortex and cerebral nuclei, as well as some from the hypothalamic preoptic area, which are derived from the developmental subpallium (1051 clusters)
**TH-EPI-Glut**	Glutamatergic neurons from thalamus and epithalamus (i.e., medial and lateral habenula) (148 clusters)
**WholeBrain**	All cells in the whole mouse brain (5322 clusters)

Table 1. Neighborhoods descriptions.

Description of cells belonging to each neighborhood group.

Table 2. Region acronyms.

Allen Mouse Brain Common Coordinate Framework (CCF) acronyms.

Htr	Defining Feature
Htr1a	Expressed in an important fraction of Sero neurons of the raphe and some HPF excitatory neurons
Htr1b	Expressed in many inhibitory striatal neurons and Sero neurons
Htr1d	Expressed in the striatum, although at much lower levels
Htr1f	Widely expressed in telencephalic structures, especially the Isocortex, with a peak in frontal olfactory structures
Htr2a	Prevalent in glutamatergic cells of the cortical subplate (CLA and EPd) and the mammillary bodies (TMd, PMd), and hippocampal interneurons
Htr2b	Rarely transcribed and is present in some neurons of the pineal gland
Htr2c	Broadly transcribed, especially in the STR, excitatory neurons of the amygdala (LA, BLA and BMA) and RSPv, OLF neurons and structures in MB, P, MY and CB
Htr3a	Uniquely observed in cortical gabaergic neurons of the 06 CTX-CGE GABA class
Htr3b	Uniquely observed in cortical gabaergic neurons of the 06 CTX-CGE GABA class
Htr4	Transcribed at high levels in the OT, excitatory cells of the hippocampus proper and DG, and Chol neurons of the TH (17 MH-LH Glut)
Htr5a	Transcribed at low levels with only one enriched cluster in the MB
Htr5b	Transcribed only in few cells, specifically in Chol neurons of the TH
Htr6	Does not feature any enriched cluster, some cells in CA3 transcribed this Htr
Htr7	Widely transcribed in subcortical structures, especially in some TH nuclei (PF, PVT, IAD and PT), the mammillary complex (MM and PMd), the lateral septal nucleus (LSv) and the fasciola cinerea of the HPF

Table 3. Htrs defining features.

Table listing the defining feature of each Htr trascription pattern.
