# Supplementary material for: Transcriptomic mapping of the 5-HT receptor landscape
Source: Patterns (N Y). 2024 Aug 29;5(10):101048. doi: 10.1016/j.patter.2024.101048 (PMC11574285; doi:10.1016/j.patter.2024.101048)
Supplement: Document S2. Article plus supplemental information [file mmc3.pdf]

Patterns

Article

Transcriptomic mapping of the 5-HT receptor landscape

Graphical abstract

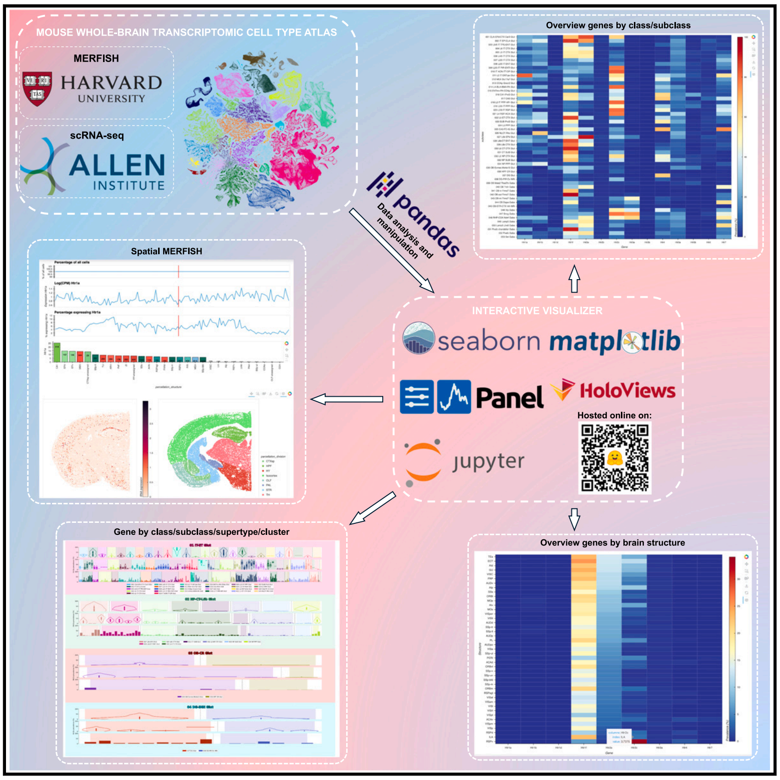

Authors

Roberto De Filippo, Dietmar Schmitz

Correspondence

roberto.de-filippo@bccn-berlin.de

In brief

Serotonin (5-HT) plays a critical role in numerous physiological and pathological processes. De Filippo and Schmitz analyzed two advanced transcriptomic atlases to map the transcription patterns of 5-HT receptors across millions of cells. Their findings reveal diverse transcription patterns of various 5-HT receptor genes. An interactive online tool is provided for custom data exploration, allowing quick, in-depth analysis across multiple grouping variables and facilitating a deeper understanding of 5-HT receptors and their implications for brain function and disorders.

Highlights

- Analysis of 5-HT-receptor single-cell mouse whole-brain transcriptomic atlases
- Extensive co-transcription points at considerable complexity at the single-cell level
- Interactive visualizer for custom exploration at different levels of granularity

De Filippo & Schmitz, 2024, Patterns 5, 101048
October 11, 2024 © 2024 The Author(s). Published by Elsevier Inc.
https://doi.org/10.1016/j.patter.2024.101048

CellPress

Patterns

CellPress
OPEN ACCESS

Article

Transcriptomic mapping of the 5-HT receptor landscape

Roberto De Filippo[1,6,*] and Dietmar Schmitz[1,2,3,4,5]
[1]Charité Universitätsmedizin Berlin, corporate member of Freie Universität Berlin, Humboldt-Universität zu Berlin, and Berlin Institute of Health, Neuroscience Research Center, 10117 Berlin, Germany
[2]German Center for Neurodegenerative Diseases (DZNE) Berlin, 10117 Berlin, Germany
[3]Charité-Universitätsmedizin Berlin, corporate member of Freie Universität Berlin, Humboldt-Universität Berlin, and Berlin Institute of Health, Einstein Center for Neuroscience, 10117 Berlin, Germany
[4]Charité-Universitätsmedizin Berlin, corporate member of Freie Universität Berlin, Humboldt-Universität Berlin, and Berlin Institute of Health, NeuroCure Cluster of Excellence, 10117 Berlin, Germany
[5]Humboldt-Universität zu Berlin, Bernstein Center for Computational Neuroscience, Philippstr. 13, 10115 Berlin, Germany
[6]Lead contact
*Correspondence: roberto.de-filippo@bccn-berlin.de
https://doi.org/10.1016/j.patter.2024.101048

THE BIGGER PICTURE Serotonin (5-HT) receptors are neurotransmitter receptor proteins with relevant roles in mood regulation, cognition, and sleep. Understanding their distribution and transcription complexity is crucial for studying overall brain function. This study leverages state-of-the-art single-cell RNA sequencing and spatial transcriptomics data to map 5-HT receptor subtypes across millions of cells in the mouse brain. The findings in this study highlight the extensive presence of these receptors, often co-transcribed within the same cell, pointing to the single-cell-level complexity of the serotonergic system, the neural system in charge of the release of neurotransmitters essential for physiological and behavioral processes such as appetite and mood regulation, cognition, and sleep. Here, we provide an interactive visualization tool to explore these data. This work sets the stage for more targeted investigations into the functional implications of 5-HT receptor distribution, with potential impacts on understanding and treating psychiatric and neurological disorders.

SUMMARY

Serotonin (5-HT) is crucial for regulating brain functions such as mood, sleep, and cognition. This study presents a comprehensive transcriptomic analysis of 5-HT receptors (Htrs) across ≈4 million cells in the adult mouse brain using single-cell RNA sequencing (scRNA-seq) data from the Allen Institute. We observed differential transcription patterns of all 14 Htr subtypes, revealing diverse prevalence and distribution across cell classes. Remarkably, we found that 65.84% of cells transcribe RNA of at least one Htr, with frequent co-transcription of multiple Htrs, underscoring the complexity of the 5-HT system even at the single-cell dimension. Leveraging a multiplexed error-robust fluorescence *in situ* hybridization (MERFISH) dataset provided by Harvard University of ≈10 million cells, we analyzed the spatial distribution of each Htr, confirming previous findings and uncovering novel transcription patterns. To aid in exploring Htr transcription, we provide an online interactive visualizer.

INTRODUCTION

Serotonin (5-HT) stands as a pivotal neuromodulator within the brain, orchestrating a diverse array of behaviors and physiological processes that include mood regulation,[1] memory,[2] sleep,[3] feeding,[4] and decision making.[5] This broad spectrum of influence is remarkably orchestrated by a limited number of 5-HT-releasing neurons located in the raphe nuclei, which extend their projections across the entire brain.[6] The intricacy of the 5-HT system is amplified by the extensive repertoire of 5-HT receptors (Htrs), most of them metabotropic receptors linked to different intracellular pathways, resulting in varied and sometimes opposing effects on neuronal excitability.[7] These receptors are categorized into 14 types spread across seven families, presenting a complex landscape of 5-HT signaling. To better grasp the impact of 5-HT release, it is crucial to accurately map the expression of each receptor throughout the brain. Historically, techniques such as immunohistochemistry,

© 2024 The Author(s). Published by Elsevier Inc.
This is an open access article under the CC BY license (http://creativecommons.org/licenses/by/4.0/).

autoradiography, *in situ* hybridization, and microarray analysis have been instrumental in shedding light on this question.[8,9] These methodologies have provided foundational insights into the brain-wide effects of 5-HT, contributing significantly to our current understanding.[10] However, the introduction of single-cell RNA sequencing (scRNA-seq) and multiplexed error-robust fluorescence *in situ* hybridization (MERFISH) has revolutionized our ability to dissect gene transcription. Unlike their predecessors, these techniques offer an unparalleled resolution, scalability, and depth of profiling, enabling detailed mapping of each Htr's transcription patterns. Such precision enhances our understanding of 5-HT's functions, setting the stage for more accurate and nuanced insights into its impact on neural circuits. To elucidate the complexities of the distribution of each Htr subtype, we utilized an scRNA-seq dataset provided by the Allen Institute,[11] incorporating the associated hierarchical clustering of cells at four nested levels. Spatial distribution data were obtained from a MERFISH dataset by Harvard University.[12] This approach revealed new patterns of Htr transcription and set a new benchmark for examining their distribution against the backdrop of existing literature, contributing to our understanding of the complex roles of 5-HT in brain function. To facilitate broader custom exploration of the Htr landscape throughout the brain, we have developed an interactive visualization tool that enables the seamless exploration of both the scRNA-seq and MERFISH datasets at different levels of granularity, providing a comprehensive view of the 5-HT receptor distribution. An online version is available at https://rdef654875678597657-5-ht-transcriptomics.hf.space.

RESULTS

Htr transcription overview

We analyzed the scRNA-seq dataset provided by the Allen Institute,[11] focusing on the transcription of Htr genes across approximately 4 million brain cells passing quality control. The scRNA-seq dataset comprehensively encompassed all known fourteen Htr subtypes. 65.84% of cells transcribed RNA of at least one Htr. To evaluate transcription, we used the same stringent threshold (log(CPM) > 3.5) (CPM = counts per million) used by the original authors to determine neurotransmitter release.[11] Prevalence of Htrs, the percentage of cells transcribing a receptor, across the entire dataset was considerably different, ranging from 0.09% of Htr3b to 34.26% of Htr1f (Figure 1A). RNA of six Htrs was found in less than 2.5% of the cells (Htr1d, Htr2b, Htr3a, Htr3b, Htr5b, and Htr6). On the other hand, RNA of Htr1f, Htr2a, and Htr2c was present in at least one of every five cells. The average amount of RNA transcription also varied across receptors (Figure S1A). Interestingly, the variation in amount of RNA shared around half (R^2 = 0.55) of the variability with the prevalence, i.e., genes that were more widespread across cells also exhibited higher transcription rates within individual cells. In addition to differences in prevalence and transcription levels, the distribution of genes across the brain also showed notable variation. This variation is highlighted by comparing the distribution patterns of the Htr1 and Htr2 families, as showcased through uniform manifold approximation and projection (UMAP) analysis (Figure 1B). The UMAP visualization was color coded according to neighborhood classification. Neighborhoods, characterized by cursory anatomical proximity and molecular signatures such as neurotransmitter release,[11] offer a condensed categorization of cell types (Figure S1B and Table S1). When looking at the UMAP distributions of individual Htrs, considerable differences were also present within each family of receptors (Figure S2). We analyzed these differences grouping cells by neurotransmitter, neighborhood, or class (Figure S1B). The cells were subdivided into four nested levels of classification (as defined previously[11]) with 34 classes, 338 subclasses, 1,201 supertypes, and 5,322 clusters. These categorizations divided cells in a highly skewed fashion (Figure S1C). For example, when looking at neurotransmitter release, three groups (Glut, Gaba, and unassigned) made up almost the totality of cells (98.47%). Expectedly, the vast majority of cells was classified as excitatory (Glut, 50.79%), and around one in every five cells was found to release GABA (20.62%). All the other neurotransmitters were found in less than 1% of the cells; in particular, 5-HT-releasing neurons (Sero) were found in only 0.04% of the cells. The pattern of Htr transcription across different neurotransmitter groups exhibited a relatively high mean Pearson correlation coefficient ($r = 0.6 \pm 0.03$). Sero and cholinergic neurons (Chol) showed the most distinct patterns of transcription with, respectively, mean $r = 0.32 \pm 0.04$ and 0.37 ± 0.05 (Figure 1C). To better evaluate the uniqueness of Htr RNA transcription per group and account for differences in amplitude, not captured by simple correlation, we employed a random forest classifier aimed at decoding the grouping variable solely from the Htrs' transcription. Overall accuracy of the model in decoding neurotransmitter was 38.55% (chance level = 10.0%). Reflecting the correlation analysis, the confusion matrix showed that Sero and Chol were among the groups with higher true-positive (TP) rate (Sero = 74.4%, Chol = 46.99%). Cells not transcribing any neurotransmitter, not exhibiting a low *r* beforehand, were, nonetheless, identified even more successfully (79.44%). Moreover, noradrenaline (Nora)- and glycine (GABA-Glyc)-releasing neurons were identified at considerable levels (Nora = 42.11% and GABA-Glyc = 42.6%). To understand the contribution of each Htr in each prediction, we calculated the mean absolute SHAP (Shapley additive explanations) values for each receptor and neurotransmitter.[13,14] The SHAP values in association with the mean prevalence enabled us to easily understand the defining features of each group. We can appreciate, for example, that the identification of Sero neurons is determined mainly by transcription of Htr1a and Chol neurons by Htr4 and Htr5b. Crucially, absence of transcription can also contribute to the classification, e.g., cells not transcribing any neurotransmitter were identified mainly by the absence of any Htr, and Nora neuron detection was guided by the unique absence of Htr4. When looking at different neighborhoods, the accuracy of the model was 40.81% (chance level = 12.5%). The model could differentiate best the NN-IMN-GC, TH-EPI-Glut, and Pallium-Glut groups (NN-IMN-GC = 72.82%, TH-EPI-Glut = 69.15%, and Pallium-Glut = 57.65%, Figure S3A). NN-IMN-GC includes all the cells not releasing any neurotransmitter; their classification was therefore expectedly influenced by absence of any Htr. On the other hand, TH-EPI-Glut cells were characterized by the unique combination of high transcription of Htr7 and low transcription of Htr2a and Htr4. Pallium-Glut cells instead exhibited relatively low levels of Htr2c and Htr7. Notably, Htr7 and Htr1f seemed

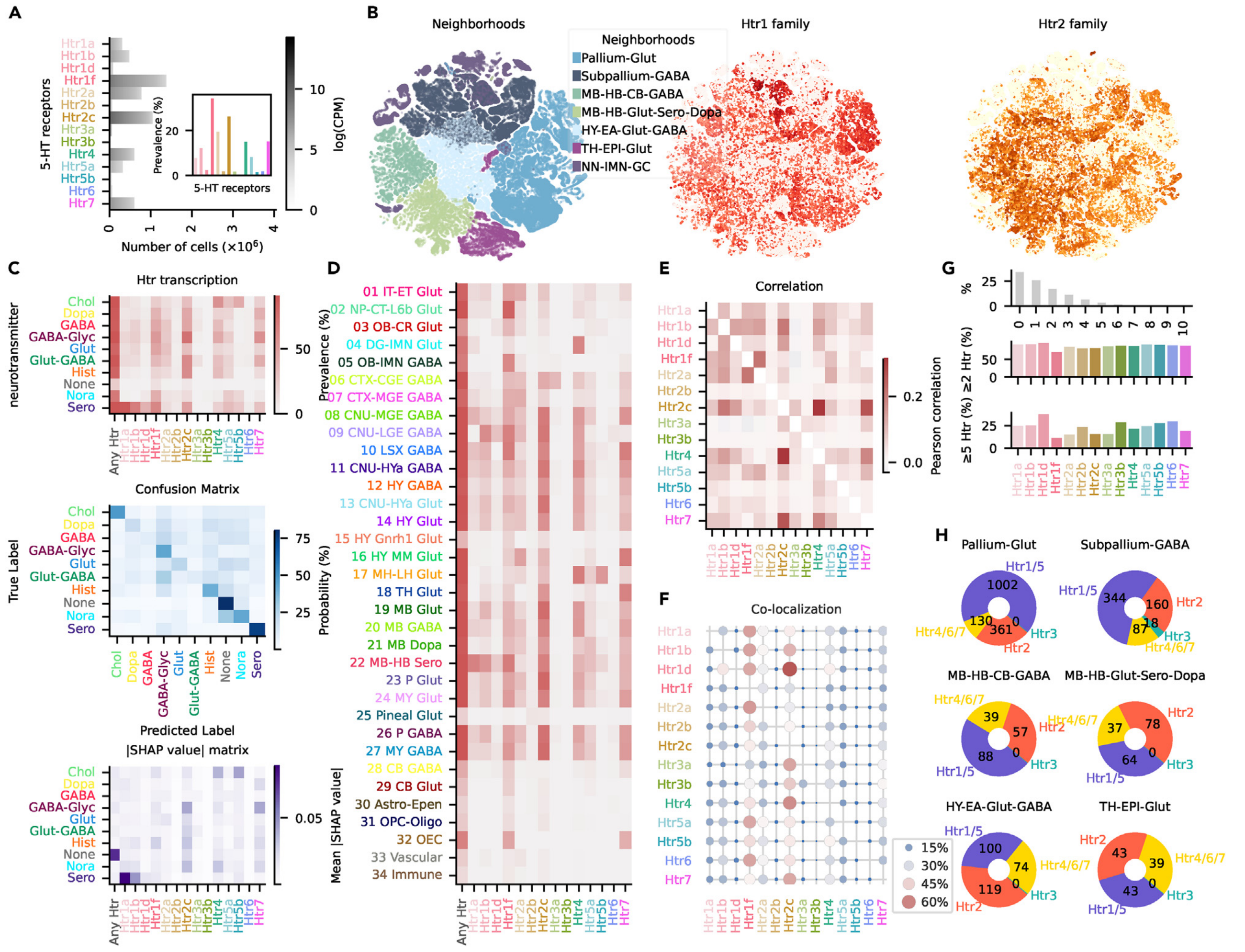

Figure 1. Overview of Htr translation

(A) Barplot showing absolute number of cells transcribing each Htr. Amount of expression is represented in grayscale; no threshold is applied. Inset shows the prevalence of each Htr using a threshold set at log(CPM) > 3.5 and same color coding.

(B) UMAP representation color coded by neighborhood metadata (left), Htr1 (middle), and Htr2 (right) transcription.

(C) Htr expression prevalence in cells grouped by neurotransmitter release (top). Confusion matrix of the multi-label random forest classifier showing True label on y axis and predicted label on x axis (middle). Matrix of absolute SHAP values for each group and receptor (bottom). Glut, glutamate; GABA, γ-aminobutyric acid; Glut-GABA, glutamate and γ-aminobutyric acid; Dopa, dopamine; None, no specific neurotransmitter; GABA-Glyc, γ-aminobutyric acid and glycine; Chol, acetylcholine (cholinergic); Hist, histamine; Sero, serotonin; Nora, norepinephrine.

(D) Htr expression prevalence in cells grouped by class.

(E) Htr expression correlation matrix.

(F) Htr co-localization matrix. Each dot represents the percentage of co-localization of gene on x axis in cells transcribing gene on y axis.

(G) Percentage of cells transcribing the number of Htrs on the x axis (top). Percentage of cells transcribing the gene on x axis transcribing at least another Htr gene (middle) or at least four other Htrs (bottom).

(H) Pie charts representing the main pathway activated by 5-HT in each neighborhood. Principal effector was identified by summing the amount of RNA belonging to receptor of the same family in each cell. Each number represents the number of cells in thousands.

to follow opposite gradients across neighborhoods. Across classes, differences in Htr transcription were even more striking (Figure 1D). Five groups could be identified with a TP rate >40%: 04 DG-IMN Glut, 09 CNU-LGE GABA, 17 MH-LH Glut, 22 MB-HB Sero, and 34 Immune (Figure S3B). 04 DG-IMN Glut were characterized by high transcription of Htr4, as shown previously,[15] and absence of the usually prevalent Htr2c. 09 CNU-LGE GABA cells showed high Htr1b and low Htr7/Htr1a; 17 MH-LH Glut exhibited high levels of Htr5b and Htr4; 22 MB-HB Sero, mirroring the results showed by Sero neurons, were characterized by high levels of Htr1a; and 34 Immune cells were identified by absence of any Htr transcription. The exclusive use of Htr transcription pattern reached an impressive 17.85% accuracy in decoding classes (chance level = 2.941176470588235%). Correlation between Htr transcription across the totality of cells ranged from −0.03 (Htr1f-Htr3a) to 0.311 (Htr4-Htr2c). Considerable correlation was also found for the Htr7-Htr2c (r = 0.264) and Htr1f-Htr2a (r = 0.212) pairs (Figure 1E). Interestingly, correlation patterns were not stable across neighborhoods (Figure S4A). For example, Pallium-Glut

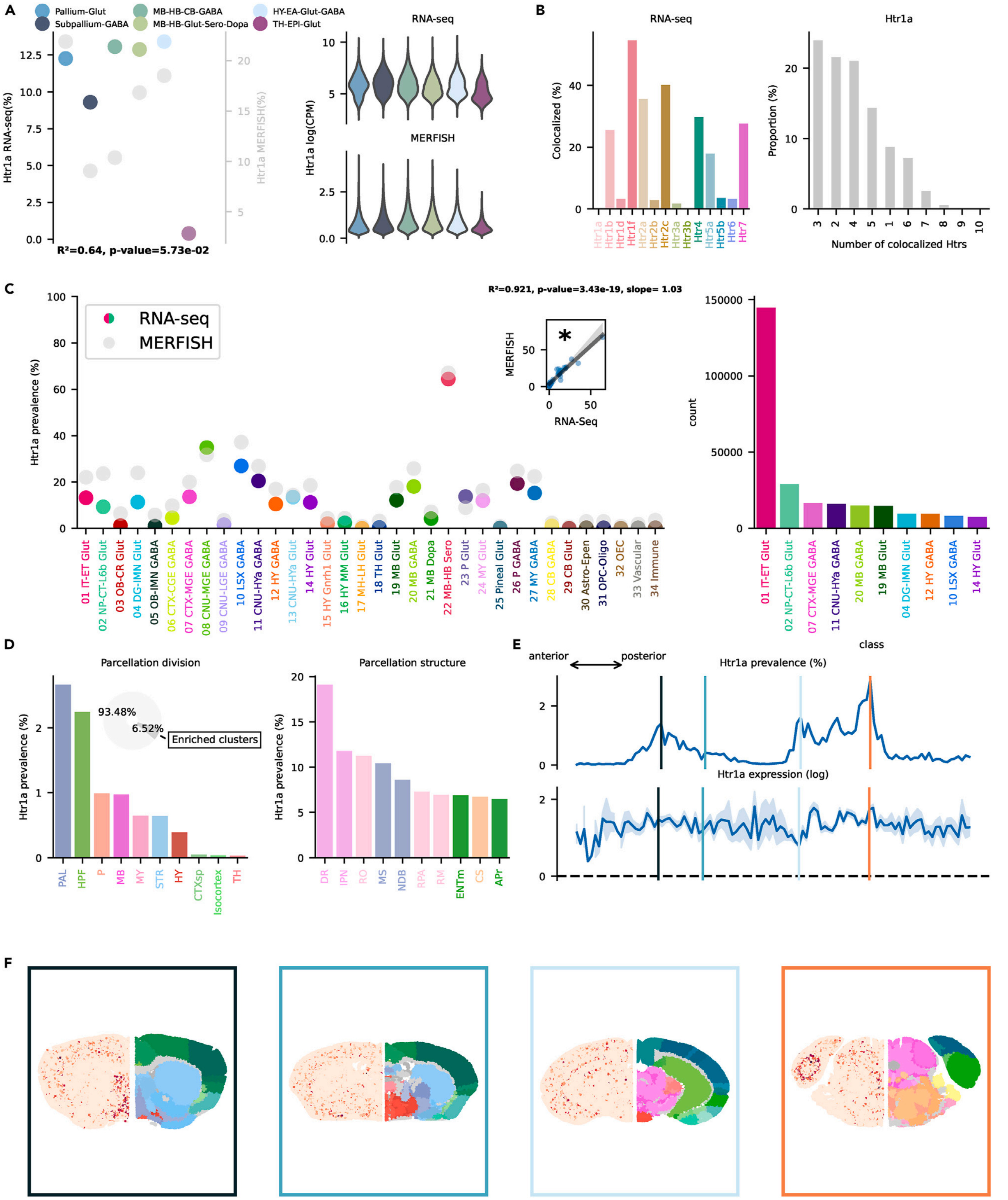

Figure 2. Htr1a transcription

(A) On the left, dotplot representing Htr1a prevalence across neighborhoods with squared Pearson correlation coefficient (R^2) between scRNA-seq and MERFISH datasets. On the right, violin plots representing the amount of Htr1a RNA detected using scRNA-seq (top) and MERFISH (bottom).

(B) Amount of co-localization with each Htr by cells expressing Htr1a RNA in the scRNA-seq dataset (left). Number of Htr RNA detected in cells expressing Htr1a RNA in the scRNA-seq dataset (right).

(legend continued on next page)

exhibited a unique negative correlation between Htr4-Htr2a not visible from the analysis of the entire dataset. Of note, TH-EPI-Glut showed the highest absolute correlation across all neighborhoods, with $r = 0.609$ between Htr5b and Htr4 and a unique negative correlation between Htr4 and Htr7. To explore the underlying causes of the correlations, we analyzed co-localization (co-transcription of RNA in the same cell) between Htrs. Across the entire dataset we observed that the most transcribed genes, Htr1f and Htr2c, were regularly transcribed whenever the RNA of any other Htr was detected (Figure 1F). This was a driving factor for correlation. Looking more in detail across neighborhoods, also here we noticed important differences, mainly explainable by differential prevalence of Htrs in each neighborhood. 60.84% of Htr-transcribing cells exhibited RNA of at least two Htrs; therefore, only in a minority of cases a cell was found to transcribe uniquely one Htr (25.78% of the totality of cells, Figure 1G). Surprisingly, 7.4% of Htr-transcribing cells were found to exhibit RNA of at least five other Htrs. The extensive transcription of different Htr families within the same cell indicates the complexity of the 5-HT system even at the single-cell dimension. To facilitate an understanding of the downstream cellular effects of 5-HT, we aggregated receptors according to their main intracellular effector. We aggregated Htr1 and Htr5 due to their inhibitory effect (cAMP decrease) and Htr4, Htr6, and Htr7 because of the shared downstream effect of increasing cAMP. Htr2 is the only one that causes a Ca^{2+} increase, while Htr3 is the only ionotropic receptor. For each cell we determined the principal pathway activated by 5-HT by analyzing the detected RNA levels for each Htr, grouping them by intracellular effector and selecting the top ranked. We grouped the results by neighborhood, informed by the differential Htr transcription (Figure 1H). Ht3 was present only in a small minority of subpallium inhibitory neurons. In the telencephalon, the absolute majority of both Pallium-Glut and Subpallium-Gaba cells were linked to Htr1/5, and around one-quarter of cells featured Htr2 as primary effector. Subcortical cells exhibited a more balanced partition without any absolute majority and a considerable presence of Htr4/6/7. In the following sections we will take a deeper look at Htrs grouped by intracellular effector. We will take advantage of the information provided by the MERFISH dataset of Zhang et al.[12] to analyze in detail their spatial distribution. The MERFISH dataset contains information about only 9 of the 14 Htr genes present in the scRNA-seq dataset. To overcome this limitation and try to pinpoint the spatial location of all genes, we first identified in the scRNA-seq dataset clusters highly enriched in the selected gene, i.e., to be classified as enriched at least 70% of cells in a given cluster must express the selected Htr gene. Taking advantage of the clustering label integration between the scRNA-seq and MERFISH datasets,[12] we could identify the spatial distribution in the MERFISH dataset of cells belonging to enriched clusters defined using the scRNA-seq dataset. This approach worked in all cases where enriched clusters could be identified. In some cases only a minority of cells transcribing the selected Htr gene belonged to enriched clusters. For this reason we offer the option to bypass the scRNA-seq enriched cluster calculations and directly view the prevalence of all cells transcribing the selected gene in the MERFISH dataset using the interactive visualizer (see "Spatial MERFISH" and "Overview genes by brain structure" dashboards, the latter with "Data source selector" = "MERFISH only").

Htr1 and Htr5

Receptors belonging to these two families have an inhibitory effect on the host cell: they are coupled to G_i and cause a downstream decrease of cAMP and activation of GIRK channels.[16,17] Some Htr1a agonists are currently used as anxiolytics[18] and antidepressants.[19–21] The link between 5-HT imbalances, 5-HT1A activation, and depression is, however, controversial and has been called into question.[22] Htr1b and Htr1d agonists, like triptans, are effective in treating migraine.[23] Htr1a RNA has a stable prevalence of $\approx$10% across neighborhoods in the scRNA-seq dataset, with virtual absence in the TH-EPI-Glut group (Figure 2A). This receptor seems to play a role in learning and cognitive flexibility.[24] Htr1a co-localized most frequently with Htr1f, Htr2c, and Htr2a (Figure 2B) and only in a minority of cases was transcribed alone (<10%). Transcription across classes was highly correlated between the scRNA-seq and MERFISH datasets (Figure 2A) and showed a good correspondence in absolute values; this was the case for the majority of other Htrs. Highest transcription per class was found in Sero neurons of the mid- and hindbrain (class 22 MB-HB Sero, Figure 2C); nonetheless, cortical excitatory neurons (01 IT-ET Glut), like in most Htrs, contained the highest absolute number of cells transcribing the receptor. Subclasses located in the hippocampus (HPF, see Table S2 for a list of acronyms) contained most of the cortical cells transcribing Htr1a (see interactive visualizer, "Overview genes by class/subclass," "Class selector" = "01 IT-ET Glut," "Group by" = "subclass"). Only 6.52% of Htr1a transcribing cells were contained in enriched clusters, indicating a relatively low importance of this receptor in the clustering algorithm used by Yao et al.[11] Looking at the spatial distribution across divisions, e.g., high-level regional areas, the highest prevalence was found in the pallidum (PAL) and HPF (Figure 2D). At a more granular level, five of the top ten structures by prevalence belonged to the raphe nuclei: dorsal nucleus raphe (DR), nucleus raphe obscurus (RO), nucleus raphe pallidus (RPA), nucleus raphe magnus, and superior central nucleus raphe (CS). The high levels of Htr1a transcription in the raphe nuclei is reflection of the high prevalence in Sero neurons outlined beforehand; the raphe nuclei, indeed, contains the vast majority of Sero neurons of the brain. The hippocampal structures exhibiting higher prevalence were the medial entorhinal cortex (ENTm) and the area

(C) Prevalence of Htr1a RNA across all classes of cells in scRNA-seq and MERFISH datasets. Inset represents the linear regression between the two datasets. On the right, absolute number of cells expressing Htr1a RNA in the scRNA-seq dataset by class, ranked in descending order (top ten).

(D) Ranked prevalence of Htr1a RNA across divisions (left) and structures of enriched clusters found in the scRNA-seq dataset in the MERFISH dataset (right). Inset represents the proportion of cells expressing Htr1a RNA that belongs to enriched clusters.

(E) (Top) Prevalence of cells from enriched clusters across the anteroposterior axis, identified in the scRNA-seq dataset and cross-referenced in the MERFISH dataset. (Bottom) Average amount of RNA expression found in enriched clusters cross-referenced in the MERFISH dataset.

(F) Expression of Htr1a RNA detected by MERFISH in four representative slices. Border color represents the position on the anteroposterior axis.

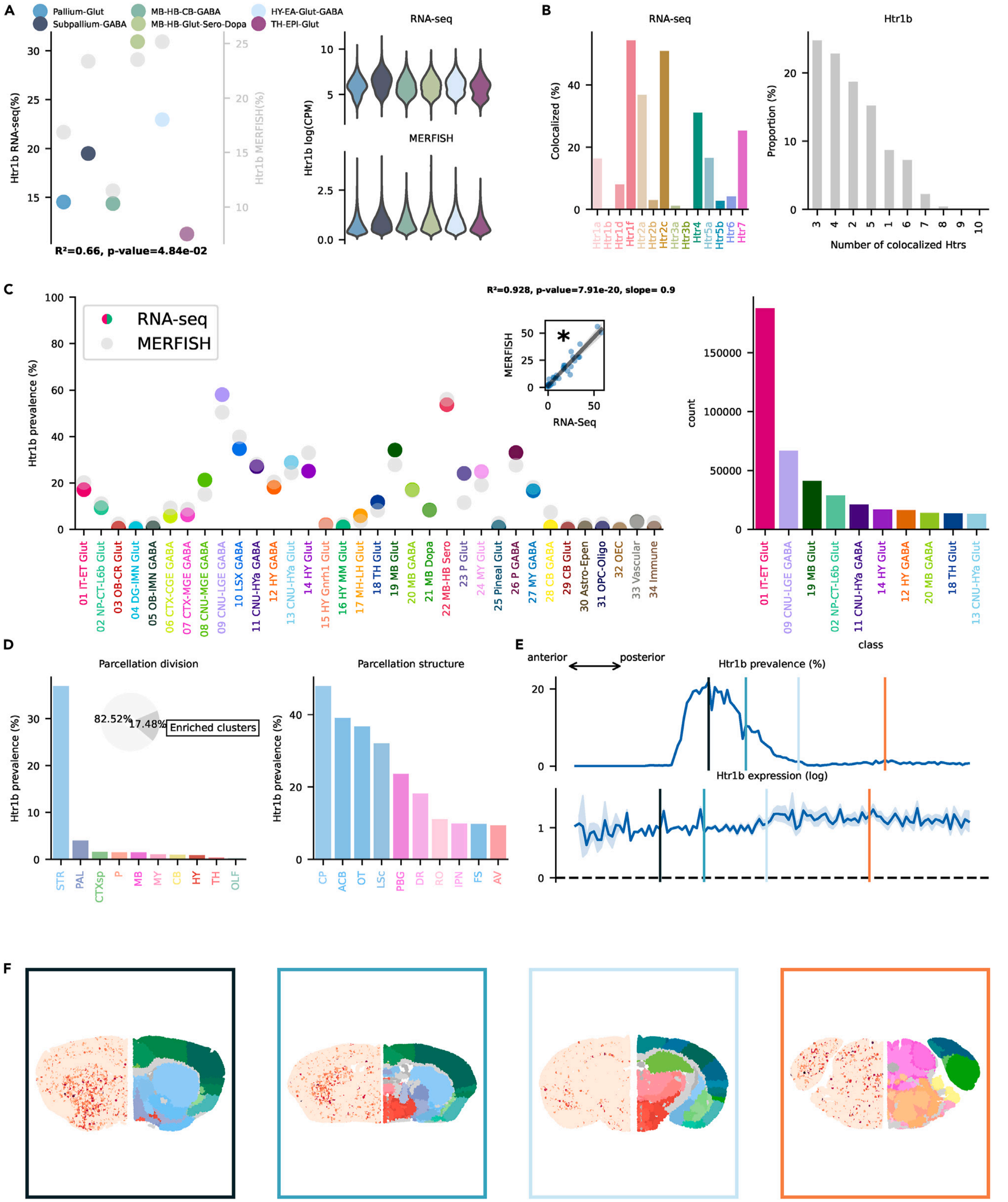

Figure 3. Htr1b transcription

(A) On the left, dotplot representing Htr1b prevalence across neighborhoods with squared Pearson correlation coefficient (R^2) between scRNA-seq and MERFISH datasets. On the right, violin plots representing the amount of Htr1b RNA detected using scRNA-seq (top) and MERFISH (bottom).

(B) Amount of co-localization with each Htr by cells expressing Htr1b RNA in the scRNA-seq dataset (left). Number of Htr RNA detected in cells expressing Htr1b RNA in the scRNA-seq dataset (right).

(legend continued on next page)

prostriata (APr), while the medial septum nucleus (MS) and the diagonal band nucleus (NDB), two structures linked to generation of theta waves[25] and containing Chol neurons, contributed substantially to the transcription in PAL. Notably, all these results confirm previous reports of Htr1a expression in the raphe,[26,27] ENTm,[28,29] and MS.[30] Levels of transcription were stable across the anterior-posterior axis, as in most other Htrs (Figures 2E and 2F). Htr1b is involved in social memory in mouse.[31] Htr1b exhibited a more diverse pattern of transcription across neighborhoods (Figure 3A) ranging from 10% to 30%. Highest prevalence was observed in the MB-HB-Glut-Sero-Dopa group (glutamatergic, serotonergic, and dopaminergic neurons located in midbrain and hindbrain). Co-localization showed a similar pattern compared to Htr1a (Figure 3B), with only a minority of cells transcribing Htr1b alone (<10%). Looking at transcription across classes, the 09 CNU-LGE GABA class showed the highest prevalence (58.06%) closely followed by 22 MB-HB Sero (53.73%) (Figure 3C). High transcription in 09 CNU-LGE GABA was in sharp contrast to Htr1a that showed only minimal transcription in this class (1.61%). Also in this case, 01 IT-ET Glut exhibited the highest absolute number of Htr1b transcribing cells, specifically in a subclass of the nucleus of the lateral olfactory tubercle (NLOT; see interactive visualizer, "Overview genes by class," "Class selector" = "01 IT-ET Glut," "Group by" = "subclass"). 17.48% of Htr1b transcribing cells belonged to enriched clusters, and the striatum (STR) showed an impressive high prevalence with >30% (Figure 3D), in line with previous reports.[32,33] Caudoputamen (CP), nucleus accumbens (ACB), olfactory tubercle (OT), lateral septal nucleus (LSc), and the parabigeminal nucleus (PBG) all exhibited a prevalence of >20%. DR and RO of the raphe nuclei also exhibited considerable prevalence. Similarly to Htr1a, Htr1b seems to be specifically linked to Sero neurons; in line with this, they have been reported to mediate self-inhibition in these neurons.[34] Distribution across the anteroposterior axes reflected the high prevalence in STR (Figures 3E and 3F). Htr1d was transcribed at a much lower level, never exceeding 7% prevalence in any neighborhood (Figure S5A). It co-localized at highest levels with Htr2c and Htr1f (Figure S5B) and only rarely was transcribed alone (<5%). Similarly to Htr1b, transcription was highest in 09 CNU-LGE GABA and 22 MB-HB Sero (Figure S5C). Notably, 09 CNU-LGE GABA exhibited the highest absolute number of cells surpassing 01 IT-ET Glut. Only a small minority of Htr1d transcribing cells belonged to enriched clusters (2.08%). The paraventricular nucleus of the thalamus (parataenial nucleus [PT] and paraventricular nucleus [PVT]) showed the highest prevalence at only >4% (Figures S5D–S5F). Htr1f, surprisingly, showed the highest levels of transcription of all Htrs in the scRNA-seq dataset. Highest prevalence was found in the pallium and subpallium groups (Figure 4A), reaching ≈50%. Other groups showed a prevalence of 30%–40% with TH-EPI-Glut at ≈20% (Figure 4A). Htr1f was found to co-localize the most with Htr2a and Htr2c (Figure 4B). In 30% of cases Htr1f was the only Htr transcribed in a cell, and co-localization decreased linearly with the number of co-transcribed Htrs (Figure 4B). Notably, the slope of the linear regression between values provided by scRNA-seq and MERFISH was significantly lower, pointing to a difference in absolute prevalence per class (Figure 4C). This difference can be imputed to the different technique employed (see https://community.brain-map.org/t/consistent-difference-in-expression-between-zhuang-and-zeng-merfish-datasets/2604/2). The two datasets are, however, still highly correlated, with 66% shared variability. This was the case also for Htr2a, Htr2c, and Htr4. Htr1f was broadly transcribed across almost all classes, including some non-neuronal cells. Pineal gland cells were a notable exception. In absolute numbers, cortical glutamatergic cells showed the highest transcription. Various subclasses located in layer 5, claustrum (CLA), and HPF exhibited prevalence >50% (see interactive visualizer, "Overview genes by class," "Class selector" = "01 IT-ET Glut," "Group by" = "subclass"). Spatial distribution showed a peculiarly asymmetric pattern with transcription concentrated in the most anterior regions. Highest transcription was observed in STR, olfactory areas (OLF), and the cortical subplate (CTXsp), reaching >20% (Figure 4D). Specifically, the highest transcription was observed in nucleus accumbens (ACB) and olfactory tubercle (OT), similarly to Htr1b. The accessory olfactory bulb (AOB) was the OLF structure with the highest prevalence. CLA and the endopiriform nucleus (EPd), on the other hand, were the CTXsp structures exhibiting the highest prevalence. Interestingly, in the CTXsp, transcription in Glut and Gaba neurons was anticorrelated. High prevalence in Glut neurons corresponded to lower prevalence in Gaba and vice versa. In CLA and EPd, Htr1f was transcribed mainly in Glut neurons while in the amygdala (LA, basolateral amygdalar nucleus [BLA], basomedial amygdalar nucleus [BMA]) predominantly in Gaba neurons (see interactive visualizer, "Overview genes by brain structure," "Division selector" = "CTXsp," "Neurotransmitter selector" = "Glut" vs. "GABA"). Isocortex and HPF also exhibited considerable transcription both in excitatory and inhibitory neurons. The amount of RNA transcription per cell was not linear, with a clear peak in the frontal olfactory areas (Figures 4E and 4F). High transcription of Htr1f in this region was previously observed using immunohistochemistry.[35] The broad transcription of Htr1f observed in the scRNA-seq dataset across the entire telencephalon is in line with earlier reports.[36] Neither Htr5a nor Htr5b were included in the MERFISH dataset; therefore, we do not have any direct spatial visualization of their transcription. Htr5a was transcribed at 8%–16% prevalence across all neighborhoods (Figure S6A) and co-localized the most with Htr1f, Htr2c, and Htr2a (Figure S6B). Transcription was broadly distributed across many classes, although only at lower levels compared to other Htrs (Figure S6C). Only one

(C) Prevalence of Htr1b RNA across all classes of cells in scRNA-seq and MERFISH datasets. Inset represents the linear regression between the two datasets. On the right, absolute number of cells expressing Htr1b RNA in the scRNA-seq by class, ranked in descending order (top ten).

(D) Ranked prevalence of Htr1b RNA across divisions (left) and structures of enriched clusters found in the scRNA-seq dataset in the MERFISH dataset (right). Inset represents the proportion of cells expressing Htr1b RNA that belongs to enriched clusters.

(E) (Top) Prevalence of cells from enriched clusters across the anteroposterior axis, identified in the scRNA-seq dataset and cross-referenced in the MERFISH dataset. (Bottom) Average amount of RNA expression found in enriched clusters cross-referenced in the MERFISH dataset.

(F) Expression of Htr1b RNA detected by MERFISH in four representative slices. Border color represents the position on the anteroposterior axis.

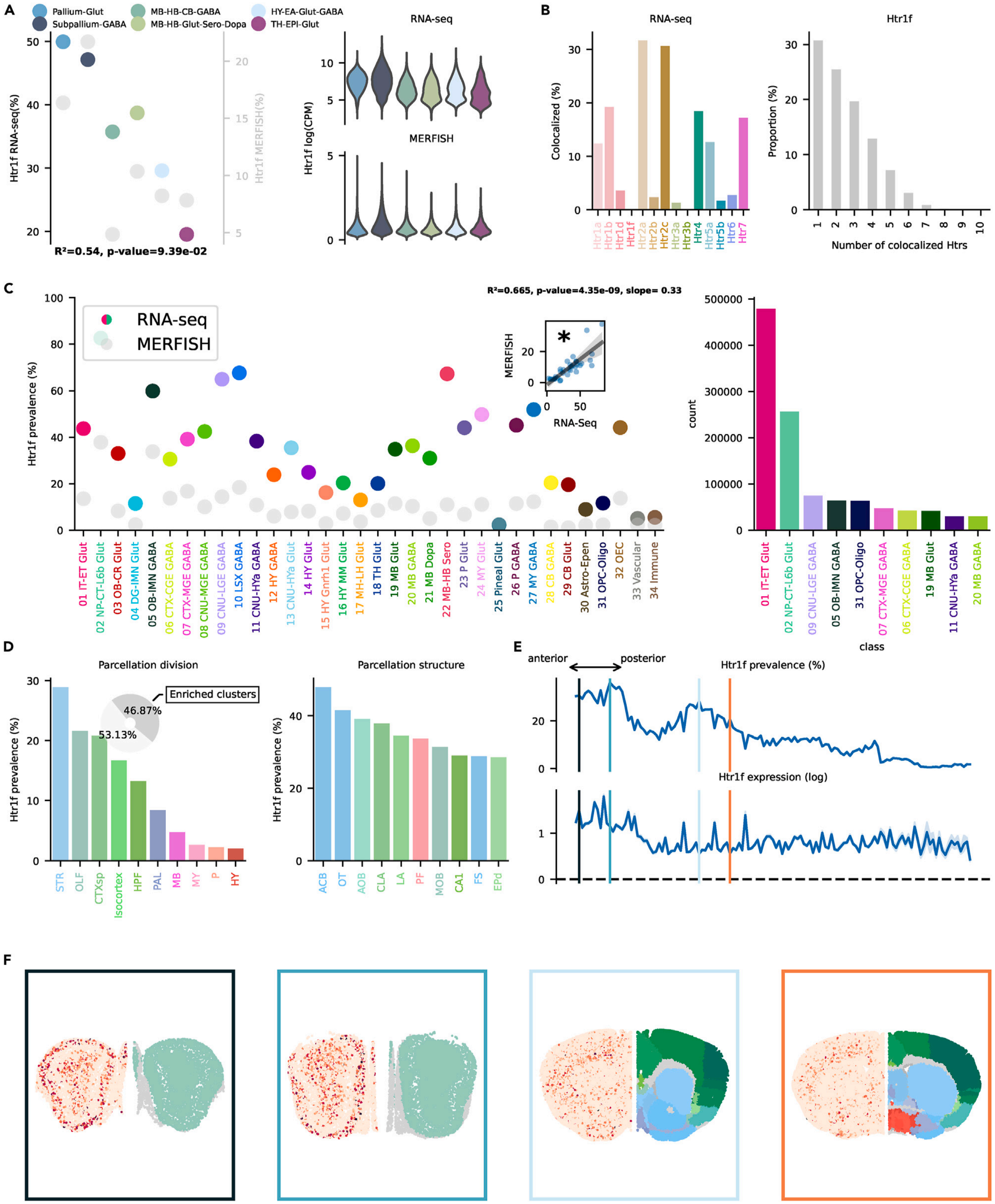

Figure 4. Htr1f transcription

(A) On the left, dotplot representing Htr1f prevalence across neighborhoods with squared Pearson correlation coefficient (R^2) between scRNA-seq and MERFISH dataset. On the right, violin plots representing the amount of Htr1f RNA detected using scRNA-seq (top) and MERFISH (bottom).

(B) Amount of co-localization with each Htr by cells expressing Htr1f RNA in the scRNA-seq dataset (left). Number of Htr RNA detected in cells expressing Htr1f RNA in the scRNA-seq dataset (right).

(legend continued on next page)

CellPress
OPEN ACCESS

cluster was considered enriched with Htr5a in the entire scRNA-seq dataset: 3453 PAG-PPN Pax5 Sox21 Gaba. This cluster was located mainly in the midbrain reticular nucleus (RR, Figures S6D and S6E). Htr5b was transcribed at a lower level across neighborhoods (Figure S7A), with a maximum of ≈5% in TH-EPI-Glut. Surprisingly, even if their overall prevalence was much lower than that of Htr5a, ten clusters were found to be enriched in Htr5b. This receptor was transcribed at considerable levels only in the 17 MH-LH Glut class (≈50% prevalence). This was reflected by high levels of transcription in the medial habenula (MH, Figures S7D and S7E), a structure involved in the response to stress and fear.[25,37–39] Some transcription was also evident in the posterior part of the brain, specifically in the inferior olivary complex (IO), driven by a single subclass, 253 IO Fgl2 Glut (see interactive visualizer, "Overview genes by class/subclass," "Class selector" = "24 MY Glut," "Group by" = "subclass"), and some structures populated by Sero neurons.

Htr2

The Htr2 family is mainly linked to Gq/11 and causes depolarization by increasing intracellular Ca^{2+}. Htr2a antagonists, such as atypical antipsychotics (e.g., clozapine and risperidone), are currently used in treating schizophrenia and other psychiatric disorders. Htr2c antagonists are being explored for their potential in treating obesity and metabolic disorders.[40,41] Htr2a, instrumental in mediating the effects of psychedelics[42] and involved in learning and memory,[43,44] is found across the brain, with highest prevalence in telencephalic neighborhoods, Pallium-Glut, and Subpallium-Gaba (Figure 5A). Co-localization was highest with Htr1f and Htr2c (Figure 5B). Highest transcription (≈40%) was found in 01 IT-ET Glut, 07 CTX-MGE GABA, and 16 HY-MM Glut classes (Figure 5C). Interestingly, somatostatin (Sst) neurons belonging to 07 CTX-MGE GABA, while exhibiting a relatively low prevalence at the subclass level, contained various clusters with >70% prevalence[45] (see interactive visualizer, "Overview genes by class/subclass," "Class selector" = "07 CTX-MGE GABA," "Subclass selector" = "053 Sst Gaba," "Group by" = "cluster"). Htr2a was also prevalent across many other classes across the whole brain. 01 IT-ET Glut exhibited by far the highest absolute number of neurons transcribing Htr2a, specifically in subclasses of layer 5 and CLA, resembling Htr1f (see interactive visualizer, "Overview genes by class/subclass," "Class selector" = "01 IT-ET Glut," "Group by" = "subclass"). CTXsp showed the highest prevalence, reaching >12% (Figure 5D). Isocortex and STR exhibited both ≈5% prevalence. At a structure level, two structures belonging to the mammillary complex (dorsal premammillary nucleus [PMd] and tuberomammillary nucleus [TMd]) were in the top ten by prevalence. The mammillary complex has been linked to Alzheimer's disease[46] and memory.[47] CLA and the EPd showed the highest absolute prevalence. Interestingly, CLA has been proposed to play an important role in mediating the effects of psychedelic compounds.[48] Prevalence in the STR was driven by the small bed nucleus (BA), a structure important for the integration of limbic and environmental information.[49] Htr2a transcription in CLA and mammillary complex is in line with a previous report in monkey.[50] Prevalence of Htr2a was highest in frontal regions of the brain, decaying linearly to virtual absence in the cerebellum (Figures 5E and 5F). Htr2b was found only in a minority of neurons and was not included in the MERFISH dataset. No cluster was found to be enriched with Htr2b. Interestingly, neurons belonging to the Pineal Glut class showed the highest prevalence at 7.34% (Figure S8C). Htr2c plays a role in feeding and impulsive behavior.[51] Htr2c was found at highest prevalence in the MB-HB-Glut-Sero-Dopa and Hy-EA-Glut-Gaba neighborhoods (Figure 6A). Apart from Pallium-Glut, its prevalence was always >40%. Co-localization was highest with Htr1f, Htr4, and Htr7 (Figure 6B). Transcription was broadly distributed across many different classes, especially subcortically (Figure 6C). Many classes exhibited a >60% prevalence. As usual, cortical excitatory neurons exhibited the highest absolute number of cells transcribing Htr2c. Some subclasses in OLF, amygdala, and retrosplenial cortex (RSP) exhibited >80% prevalence (see interactive visualizer, "Overview genes by class/subclass," "Class selector" = "01 IT-ET Glut," "Group by" = "subclass"). The majority of cells expressing Htr2c RNA belonged to enriched clusters. Highest prevalence was found in STR. Similarly to Htr1b, ACB, CP, and OT exhibited the highest prevalence (Figures 6D–6F). Isocortex prevalence derived from the unique transcription in excitatory neurons of the ventral part of the RSP, curiously the area with lowest transcription of Htr1f, which was otherwise highly prevalent in all other cortical regions (see interactive visualizer, "Overview genes by brain structure," "Division selector" = "Isocortex," "Neurotransmitter selector" = "Tot"). Htr2a RNA was also minimally expressed in this specific area. High prevalence was observed also in excitatory neurons of the anterior olfactory nucleus (AON), piriform area and piriform amygdalar area), and amygdala (LA and BLA). Htr2c RNA was found across a variety of structures also in the MB (not in Sero neurons), pons (P), medulla (MY), and cerebellum (CB).

Htr4, Htr6, and Htr7

These receptors are all connected to Gs,[17] leading to increasing cellular levels of cAMP. Htr4 modulation in HPF has been found to bidirectionally influence memory formation in mice.[2] Htr4, similarly to Htr2c, showed highest prevalence (>40%) in the MB-HB-Glut-Sero-Dopa and Hy-EA-Glut-Gaba groups (Figure 7A). Htr4 has been linked to mood control and anxiety.[52] It co-localized the most with Htr2c and Htr1f (Figure 7B). Transcription across classes was broadly distributed, with many

(C) Prevalence of Htr1f RNA across all classes of cells in scRNA-seq and MERFISH datasets. Inset represents the linear regression between the two datasets. On the right, absolute number of cells expressing Htr1f RNA in the scRNA-seq by class, ranked in descending order (top ten).

(D) Ranked prevalence of Htr1f RNA across divisions (left) and structures of enriched clusters found in the scRNA-seq dataset in the MERFISH dataset (right). Inset represents the proportion of cells expressing Htr1f RNA that belongs to enriched clusters.

(E) (Top) Prevalence of cells from enriched clusters across the anteroposterior axis, identified in the scRNA-seq dataset and cross-referenced in the MERFISH dataset. (Bottom) Average amount of RNA expression found in enriched clusters cross-referenced in the MERFISH dataset.

(F) Expression of Htr1f RNA detected by MERFISH in four representative slices. Border color represents the position on the anteroposterior axis.

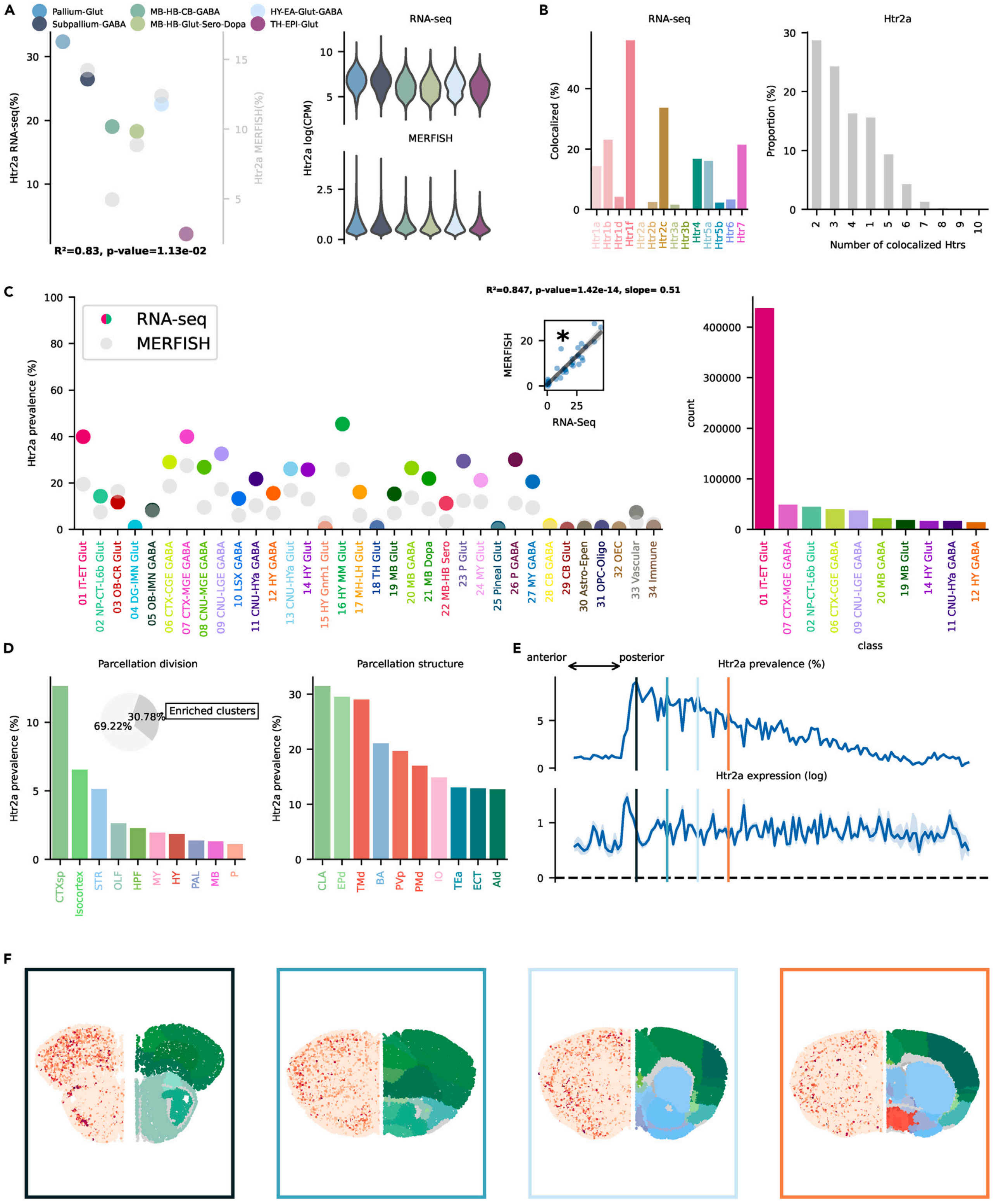

Figure 5. Htr2a transcription

(A) On the left, dotplot representing Htr2a prevalence across neighborhoods with squared Pearson correlation coefficient (R^2) between scRNA-seq and MERFISH datasets. On the right, violin plots representing the amount of Htr2a RNA detected using scRNA-seq (top) and MERFISH (bottom).

(B) Amount of co-localization with each Htr by cells expressing Htr2a RNA in the scRNA-seq dataset (left). Number of Htr RNA detected in cells expressing Htr2a RNA in the scRNA-seq dataset (right).

(legend continued on next page)

subcortical classes showing a prevalence >40% (Figure 7C). Highest prevalence was found in the 17 MH-LH Glut class, specifically in Chol-releasing neurons belonging to this class located in TH. In absolute numbers, transcription in excitatory cortical neurons was the highest, but not overwhelmingly so like in other cases, driven specifically by subclasses of CA1, CA2, CA3, and subiculum (see interactive visualizer, "Overview genes by class/subclass," "Class selector" = "01 IT-ET Glut," "Group by" = "subclass"). Spatial distribution exhibited a peculiar pattern with high prevalence in one specific structure of the STR, namely OT (Figures 7D–7F). A subclass of interneurons present in OT (060 OT D3 Folh1 Gaba) showed a >98% prevalence (see interactive visualizer, "Overview genes by class/subclass," "Class selector" = "09 CNU-LGE GABA," "Group by" = "subclass"). PAL and HPF also exhibited relatively high prevalence (≈10%). Dentate gyrus (DG) granule cells (037 DG Glut) were one of the reasons for the high prevalence in HPF. Excitatory cells of CA2, CA3, and indusium griseum (IG) also transcribed Htr4 RNA (see interactive visualizer, "Overview genes by brain structure," "Division selector" = "HPF," "Neurotransmitter selector" = "Glut" vs. "GABA"). We do not have MERFISH information about the rarely transcribed Htr6, and no enriched cluster was present in the scRNA-seq dataset. The 09 NU-LGE GABA class exhibited the highest prevalence with 7.73%; still, the absolute majority of neurons expressing the RNA of this gene were excitatory cortical neurons (Figure S9C). Conversely, Htr7 was transcribed in >10% of the totality of cells. It reached ≈60% in the TH-EPI Glut group and considerable amounts (≈40%) in MB, HB, and HY groups (Figure 8A). Colocalization was the highest with Htr2c and Htr1f (Figure 8B). Transcription was broadly distributed across classes present in HY, MB, and TH (Figure 8C). It co-localized the most with Htr2c, Htr1f, and Htr4. Htr7 was broadly transcribed across classes, especially in subcortical structures. Peak prevalence was found in 10 LSX GABA, 16 MY MM Glut, and 18 TH Glut with >60% (Figure 8C). Cortical transcription in excitatory neurons is driven primarily by subclasses in CA2 and L2 ENT (see interactive visualizer, "Overview genes by class/subclass," "Class selector" = "01 IT-ET Glut," "Group by" = "subclass"). Htr7 enriched clusters were located mainly in HY and TH (Figure 8D). At a structure level, the parafascicular nucleus (PF) and PVT of TH showed the highest prevalence (>30%).

Htr3

The Htr3 family is the only ionotropic Htr, and it causes direct excitation by allowing the influx of cations. The Htr3a subunit is required for the formation of a functional channel[53] and can form functional homopentameric receptors.[54] Heteromeric receptors containing Htr3b have an increased channel conductance and different selectivity.[55] Htr3a is transcribed almost uniquely in the Subpallium-Gaba neighborhood, with a prevalence of ≈8% (Figure 9A), specifically in the 06 CTX-CGE GABA class (Figure 9C). It is one of the few Htrs, together with Htr3b and Htr1d, that is not transcribed the most in absolute numbers in 01 IT-ET Glut. It co-localizes mainly with Htr2c and Htr7 (Figure 9B). This Htr was mainly transcribed in OLF, CTXsp, HPF, and isocortex (Figure 9D) and is most prevalent in the anterior part of the brain, although, puzzlingly, with slightly lower amount of RNA per cell (Figures 9E and 9F). Htr3b was not included in the MERFISH dataset, and no cluster was found to be enriched with this receptor. Htr3b was the least transcribed Htr gene in the entire scRNA-seq dataset. Similarly to Htr3a, its transcription was delimited to the 06 CTX-CGE GABA class (Figure S10C).

DISCUSSION

In this work we have described the main transcriptional features of each Htr across the entire brain, leveraging two datasets provided by Yao et al.[11] and Zhang et al.[12] In the scRNA-seq dataset, we found that Htrs' RNA is transcribed in around two out of every three cells and that six Htrs were transcribed in >10% of cells, with Htr1f reaching a peak of 34.26%. Htr patterns of transcription can be used to decode the identity of cells grouped by neurotransmitter, neighborhoods, and classes at a level above chance. Surprisingly, it was common to detect multiple Htrs within a single cell. This indicates the great complexity of the 5-HT system even at a cellular level. We can recapitulate our results regarding each Htr by summarizing the defining feature of each receptor (Table S3): Htr1a is transcribed in an important fraction of Sero neurons of the raphe and some HPF excitatory neurons; Htr1b is transcribed in many inhibitory striatal neurons and Sero neurons; Htr1d, similarly to Htr1b, is transcribed in the striatum, although at much lower levels; Htr1f is widely transcribed in telencephalic structures, especially the isocortex, with a peak in frontal olfactory structures; Htr2a is prevalent in glutamatergic cells of the cortical subplate (CLA and EPd) and the mammillary bodies (TMd and PMd), and hippocampal interneurons; Htr2b is rarely transcribed and is present in some neurons of the pineal gland; Htr2c is broadly transcribed, especially in the STR, excitatory neurons of the amygdala (LA, BLA, and BMA) and RSPv, OLF neurons, and structures in MB, P, MY, and CB; Htr3a and Htr3b are uniquely observed in cortical GABAergic neurons of the 06 CTX-CGE GABA class; Htr4 is transcribed at high levels in the OT, excitatory cells of the hippocampus proper and DG, and Chol neurons of the TH (17 MH-LH Glut); Htr5a is transcribed at low levels with only one enriched cluster in the MB; Htr5b is also transcribed only in few cells, specifically in Chol neurons of the TH; Htr6 does not feature any enriched cluster, and some cells in CA3 transcribed this Htr; Htr7 is widely transcribed in subcortical structures, especially in some TH nuclei (PF, PVT, IAD, and PT), the mammillary complex (MM

(C) Prevalence of Htr2a RNA across all classes of cells in scRNA-seq and MERFISH datasets. Inset represents the linear regression between the two datasets. On the right, absolute number of cells expressing Htr2a RNA in the scRNA-seq by class, ranked in descending order (top ten).

(D) Ranked prevalence of Htr2a RNA across divisions (left) and structures of enriched clusters found in the scRNA-seq dataset in the MERFISH dataset (right). Inset represents the proportion of cells expressing Htr2a RNA that belongs to enriched clusters.

(E) (Top) Prevalence of cells from enriched clusters across the anteroposterior axis, identified in the scRNA-seq dataset and cross-referenced in the MERFISH dataset. (Bottom) Average amount of RNA expression found in enriched clusters cross-referenced in the MERFISH dataset.

(F) Expression of Htr2a RNA detected by MERFISH in four representative slices. Border color represents the position on the anteroposterior axis.

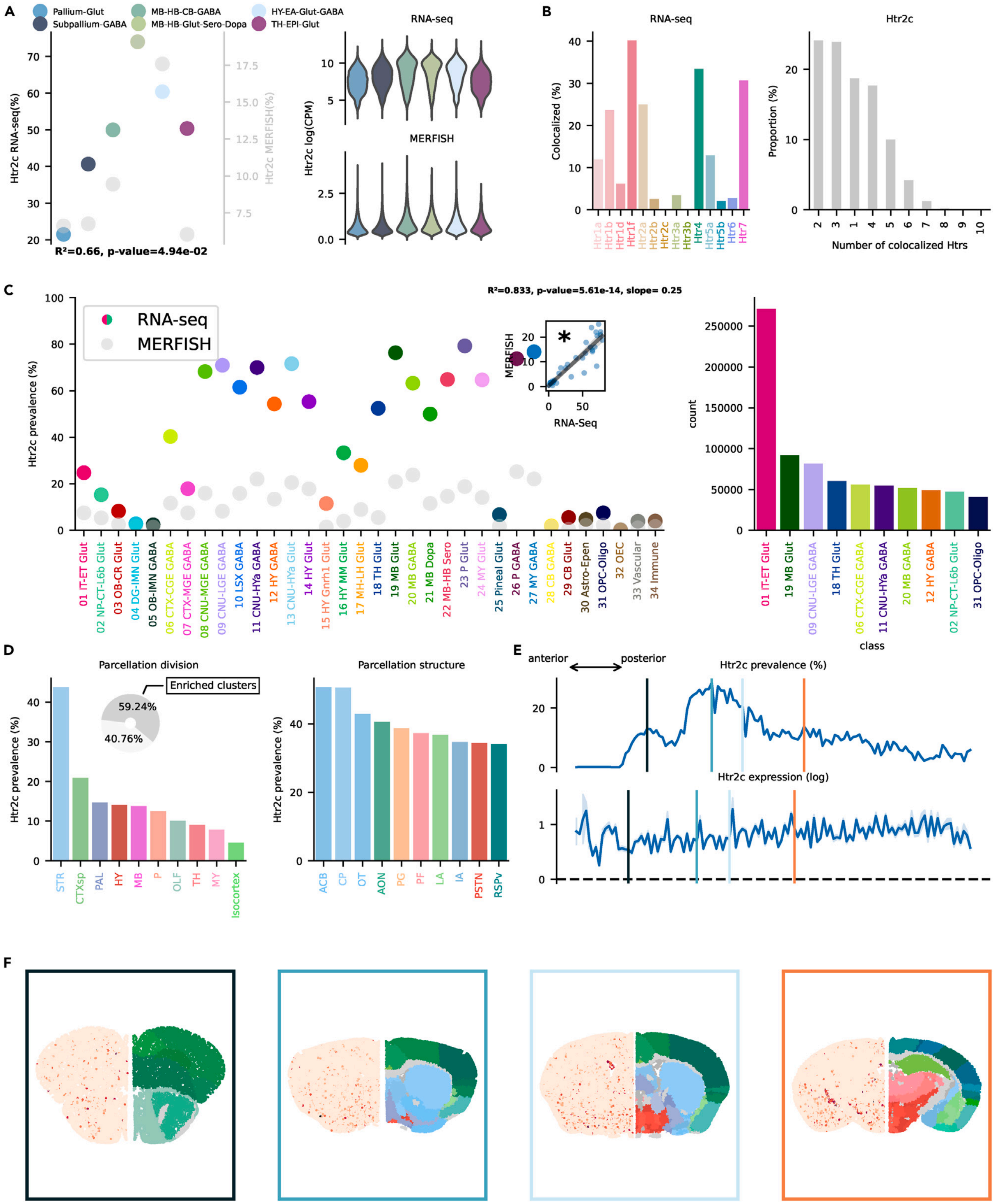

Figure 6. Htr2c transcription

(A) On the left, dotplot representing Htr2c prevalence across neighborhoods with squared Pearson correlation coefficient (R^2) between scRNA-seq and MERFISH datasets. On the right, violin plots representing the amount of Htr2c RNA detected using scRNA-seq (top) and MERFISH (bottom).

(B) Amount of co-localization with each Htr by cells expressing Htr2c RNA in the scRNA-seq dataset (left). Number of Htr RNA detected in cells expressing Htr2c RNA in the scRNA-seq dataset (right).

(legend continued on next page)

and PMd), the lateral septal nucleus (LSv), and the fasciola cinerea of the HPF. Our analysis is in no way exhaustive, and it is limited in scope by the constraints of a traditional scientific article. To bypass this limitation and, at the same time, provide the ability to explore the 5-HT transcription landscape at different depths, we provide a custom interactive visualizer. The visualizer enables the exploration of: Htr transcription in the MERFISH dataset; the prevalence of each Htr across neighborhoods, class, subclass, supertype, and clusters; an overview of Htr prevalence across classes and subclasses; and an overview of Htr prevalence across all brain divisions and structures optionally filtered by neurotransmitter release. Our entire analysis pipeline can be easily modified to enable the exploration of different families of genes. Instructions are available in the "data visualizer" section in the experimental procedures. One constraint of our study is the indirect characterization of Htrs through the detection of RNA molecules rather than direct assessment of their presence. However, this potential limitation is mitigated by the fact that mRNA levels are frequently a reliable indicator of receptor expression.[8] Conversely, while mapping receptors directly allows for precise localization, it fails to differentiate between pre- and postsynaptic expression, an important aspect of understanding receptor function and distribution. This lack of specificity becomes particularly problematic, for example in the context of Sero neurons, which have extensive projections throughout the brain and exhibit diverse autoreceptors. This complexity is underscored both in our findings and in previous research, highlighting the intricate regulatory mechanisms of 5-HT neurotransmission.[26,34] Our exploration of the Htr landscape represents a substantial advancement, contributing to our understanding of the 5-HT system's role in brain function and behavior.

EXPERIMENTAL PROCEDURES

Data preparation

We loaded the metadata and the precomputed transcription matrices ("exp" pandas dataframe) for the scRNA-seq dataset relative to all Htr genes (see "Load_data/Download_RNAseq_data.ipynb"). We also loaded the metadata relative to the "cluster_group_name" (or "neighborhood" in the text) residing originally in a different .csv file ("Find membership df" in "Figure_1.ipynb"). This information is referred to as "membership." Additionally, we loaded cell metadata information ("cell" dataframe). Each of these data structures are pandas dataframes that can be easily joined together according to the unique cell label index ("joined" dataframe). A different dataframe containing membership information is created ("joined_with_membership"); this is necessary because some cells are assigned to multiple "cluster_group_name" and therefore cause the duplication of some dataframes' rows. We used the dataframe containing "membership information" only to visualize information relative to "cluster_group_name."

The MERFISH dataset was loaded in a similar fashion (see "Load data MERFISH" in "Figure_2.ipynb"). This dataset is split into four different dataframes ("Zhuang-ABCA-1," "Zhuang-ABCA-2," "Zhuang-ABCA-3," and "Zhuang-ABCA-4") stored in a dictionary ("cell_expression"). We concatenated the four dataframes in one data structure called "data_merfish" using the "concat()" pandas method. Additionally, we used the spatial information of each cell belonging to the MERFISH dataset for the registration to the Allen Mouse Brain Common Coordinate Framework (CCF) and, subsequently, we assigned parcellations labels ("CCF registration and parcellation annotation" in "Figure_2.ipynb"). The dataframes loaded by both datasets already included all the clustering labels (class, subclass, supertype, and cluster). For details about the clustering, see the "clustering scRNA-seq data" section in Yao et al.[11]

Overview figure

This figure relies uniquely on the scRNA-seq dataset. In panel A we used a heatmap to visualize both the amount of transcription per cell and the number of cells transcribing each Htr contained in the dataset using the "exp" dataframe. In panel B we used the precomputed UMAP coordinates available in the "joined" dataframe to create a scatterplot and plotted on the color axis information about the most transcribed gene per selected family (either Ht1 or Ht2). In panel C we plotted the percentage of cells transcribing each Htr grouped by neurotransmitter release. We take advantage of pandas "Group by" function to concisely perform this computation: after grouping by the selected variable (in this case "neurotransmitter"); we apply a function called "percentage_above_threshold" to compute the percentage of cells within a group transcribing a gene above a threshold. The "percentage_above_threshold" function is defined within the "Utils.Utils.py" file. The threshold is stored in the "Utils.Settings.py" file ("threshold_expression"). The confusion matrix is computed within the "decoddddddd" function defined in Utils.Utils.py. This function uses a boolean version of the "joined" dataframe created using the same threshold ("threshold_expression"). The dataset containing boolean values for gene transcription ("joined_boolean") was filtered to include columns of interest, specifically a selector column ("sel") and a list of selected genes ("selected_genes"). The resulting dataframe was indexed by the selector column, which represented the target variable, while the remaining columns contained features corresponding to the transcription levels of various serotonin receptor genes (Htr). In this particular case, the features for classification were defined as the boolean transcription of the various 5-HT receptor genes, and the target variable was the neurotransmitter type. A random forest classifier ("RandomForestClassifier" from scokit-learn) was initialized with 200 estimators, a maximum depth of 10, balanced class weights, and parallel processing across 20 jobs. Linear models such as "LogisticRegression" and "LinearDiscriminantAnalysis" were found to underperfom the random forest classifier (respectively, 0.3768 and 0.249 accuracy vs. 0.385 for the random forest classifier, see "Test linear models" in Figure_1.ipynb). Using stratified K-fold cross-validation with 5 ("n_splits" set in Utils.Settings.py) folds, balanced accuracy scores were computed, and the mean accuracy was reported. Predictions were generated with cross-validation ("cross_val_predict" function in scikit-learn). The performance of the model was evaluated by comparing the predicted labels with the actual labels. Additionally, a comprehensive classification report was generated, providing metrics such as precision, recall, and F1 score for each class. A confusion matrix, normalized by the True labels, was also produced to visualize the model's classification performance across different neurotransmitter types. The evaluation of the model's performance was performed using scikit-learn's "balanced_accuracy_score," "classification_report," and "confusion_matrix" functions. SHAP values were calculated to interpret the feature importance of the random forest classifier. An explainer object was created using SHAP's "Tree Explainer," which was specifically designed for tree-based models. The explainer was initialized with the trained random forest classifier, and the number of parallel jobs was set to 40 to leverage computational resources effectively. The SHAP values were computed for a sample of the feature set of

(C) Prevalence of Htr2c RNA across all classes of cells in scRNA-seq and MERFISH datasets. Inset represents the linear regression between the two datasets. On the right, absolute number of cells expressing Htr2c RNA in the scRNA-seq by class, ranked in descending order (top ten).
(D) Ranked prevalence of Htr2c RNA across divisions (left) and structures of enriched clusters found in the scRNA-seq dataset in the MERFISH dataset (right). Inset represents the proportion of cells expressing Htr2c RNA that belongs to enriched clusters.
(E) (Top) Prevalence of cells from enriched clusters across the anteroposterior axis, identified in the scRNA-seq dataset and cross-referenced in the MERFISH dataset. (Bottom) Average amount of RNA expression found in enriched clusters cross-referenced in the MERFISH dataset.
(F) Expression of Htr2c RNA detected by MERFISH in four representative slices. Border color represents the position on the anteroposterior axis.

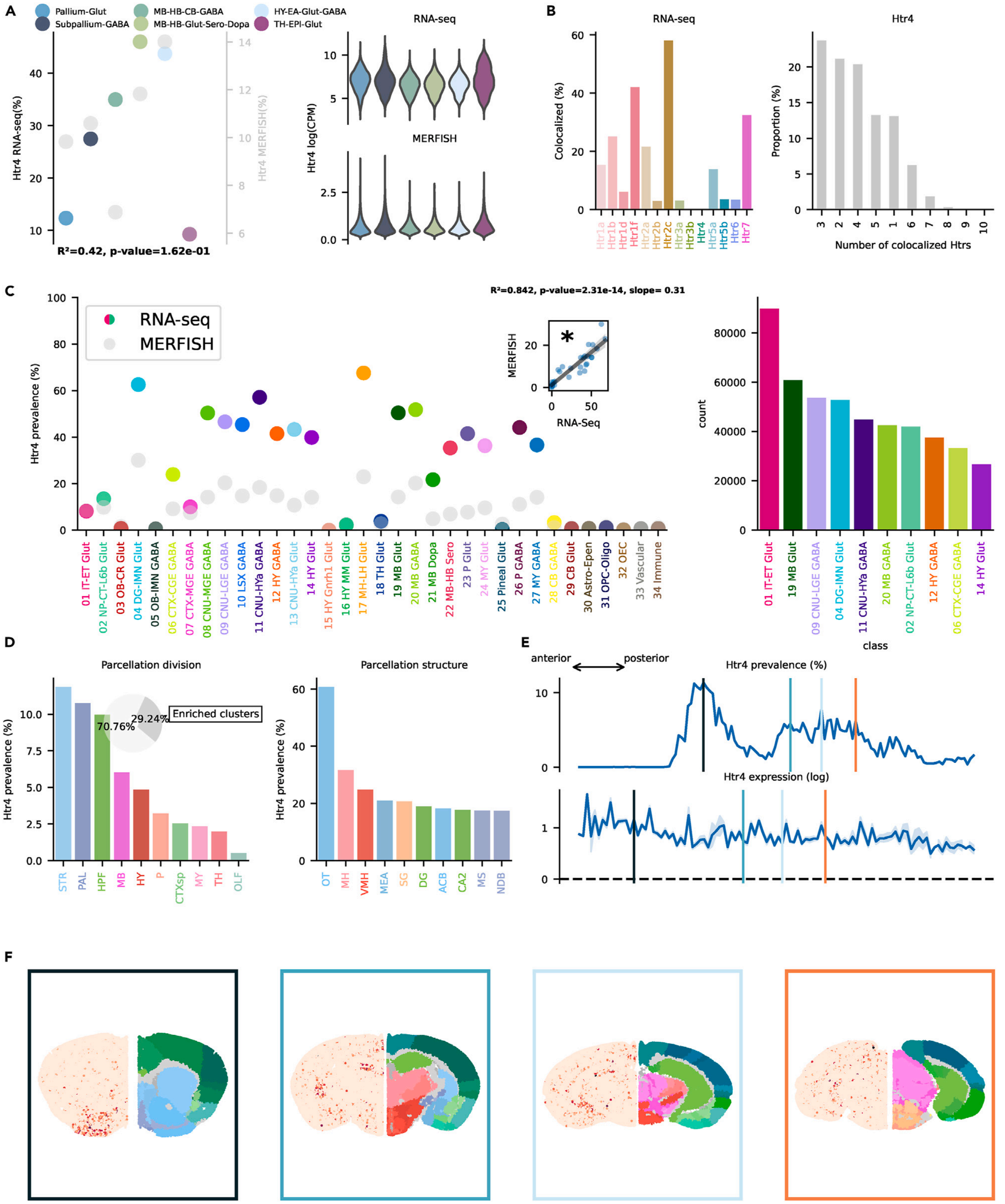

Figure 7. Htr4 transcription

(A) On the left, dotplot representing Htr4 prevalence across neighborhoods with squared Pearson correlation coefficient (R^2) between scRNA-seq and MERFISH datasets. On the right, violin plots representing the amount of Htr4 RNA detected using scRNA-seq (top) and MERFISH (bottom).

(B) Amount of co-localization with each Htr by cells expressing Htr4 RNA in the scRNA-seq dataset (left). Number of Htr RNA detected in cells expressing Htr4 RNA in the scRNA-seq dataset (right).

(legend continued on next page)

CellPress
OPEN ACCESS

10,000 observations based on class weights ("X_sample"). These values indicate the contribution of each feature to the model's predictions.

In panel D we plotted the percentage of cells transcribing each Htr grouped by class label; additional plots related to classification accuracy were computed following the instructions of the previous panel and are available as a supplemental figure. In panel E we plotted the correlation between transcription of different Htr genes by using the pandas "corr()" method. To plot the co-localization data of panel F, a dictionary named "coexp" was initialized to store the co-localization results. This dictionary would eventually hold the percentage of co-localization for each pair of genes. A nested loop was employed to iterate through each pair of selected genes, excluding a placeholder category labeled "Any Htr." For each target gene and gene to check, the following computations were performed. Co-localization calculation: for each gene pair, the boolean dataframe "joined_boolean" was used to check whether both genes were transcribed (True) in each sample. This was done using the "all(axis=1)" method, which returned True for rows where both genes were transcribed. The sum of these True values indicated the total number of samples where both genes were co-transcribed. Normalization: This sum was then normalized by dividing it by the total number of samples where the target gene was transcribed. This provided the percentage of samples where the gene pair was co-transcribed relative to the transcription of the target gene. Storing results: the computed co-localization percentage for each gene pair was stored in the coexp dictionary with the gene pair as the key. After computing the co-localization percentages for all gene pairs, the results were converted into a pandas dataframe for further analysis and visualization. The same co-localization was used in the barplots of panel G. For panel H we aggregated Htr transcription by family. These genes were grouped into four primary families: Htr1/5, summing the transcription levels of genes Htr1a, Htr1b, Htr1d, Htr1f, Htr5a, and Htr5b; Htr2, summing the transcription levels of genes Htr2a, Htr2b, and Htr2c; Htr4/6/7, summing the transcription levels of genes Htr4, Htr6, and Htr7; and Htr3, summing the transcription levels of genes Htr3a and Htr3b. These aggregated values were combined with additional columns representing neuronal classifications (class, subclass, supertype, and cluster_group_name). The columns of the resulting dataframe were labeled accordingly, and a new column ("Primary Htr family") was added. This column identified the primary serotonin receptor family for each entry by determining the family with the highest aggregated transcription.

Receptor figure

This figure relies on both the scRNA-seq and MERFISH datasets. In panel A we plot both the prevalence and the average amount of transcription of the selected gene in the two datasets. We excluded from the analysis the "NN-IMN-GC" neighborhood because of consistently low transcription across all Htr genes. For the visualization of gene transcription patterns across different "neighborhoods," we used the Seaborn "pointplot" function to illustrate the transcription levels of a given gene across various groups. The "violinplot" function was used to create violin plots of amount of transcription per group. In panel B we used the same co-localization data used in Figure 1F (scRNA-seq dataset), This barplot is a "sliced" version of that panel focusing on one receptor at the time. To visualize the number of co-localized genes (barplot on the right), we utilized a boolean dataframe ("joined_boolean") to filter for selected genes and focus on the transcription status of a particular gene. We then calculated the sum of True values (indicating gene transcription) across each row where the specific gene was transcribed. The distribution of these sums was normalized to obtain the percentage of samples exhibiting co-transcription of the genes. In panel C, on the left we repeated the same computation of panel A but using "class" as grouping variable. On the right, we plotted the raw number of cells transcribing the selected gene across different classes. We first filtered the "joined" dataframe to include only rows where the transcription level of a specific gene exceeded a defined threshold ("threshold_expression"). We then counted the occurrences of each class in this filtered dataset. The top ten classes with the highest counts were selected for visualization. Using Seaborn's barplot function, we created a barplot to display the distribution of these classes. The y axis represented the count of occurrences, while the x axis denoted the different classes. In panel D we plotted the prevalence of the selected gene in brain regions at two different hierarchical levels, "division" and "structure." Here we take advantage of the high-confidence label integration between the scRNA-seq and MERFISH dataset.[12] Each cell of the MERFISH dataset is assigned a cell-type label ("class," "subclass," "supertype," and "cluster") from the clustering of the scRNA-seq.[11] To analyze the transcription of specific genes across different brain regions and neuronal clusters, we utilized a multi-step data-processing approach. First, we calculated in the scRNA-seq the percentage of cells within each cluster transcribing the target gene above a defined threshold ("threshold_expression"), grouping the data by cluster. This allowed us to identify clusters with high gene transcription levels (>70%, "threshold_enriched_clusters" in Utils.Settings.py) in the scRNA-seq. Next, we focused on clusters with significant gene transcription, filtering the MERFISH dataset to include only cells belonging to these enriched clusters. We then computed the prevalence of cells transcribing the selected gene across different parcellation divisions and structures. This was done by normalizing the number of cells transcribing the gene in each division or structure by the total number of cells in that division or structure, expressed as a percentage. The results were visualized using barplots to illustrate the top ten parcellation divisions and structures with the highest gene transcription prevalence. Additionally, we included an inset pie chart to show the proportion of gene transcription attributable to the enriched clusters relative to the total gene transcription. This pie chart highlighted the contribution of these enriched clusters to the overall transcription of the target gene. The pie chart showed that in some cases only a minority of cells transcribing a selected gene belongs to enriched clusters and, in this case, consequentially the majority of cells will be ignored. To address this problem we include in the interactive visualizer ("Overview genes by brain structure" dashboard), a data source selector that can switch the algorithm used by the dashboard from "scRNA-seq+MERFISH" (the one described above) to "MERFISH only." This latter option computes the prevalence using solely the MERFISH dataset by simply calculating the proportion of cells transcribing the selected genes across spatial groups. In panel E, we calculated the percentage of cells within each cluster that expressed the target gene above a defined threshold ("threshold_expression"), allowing us to identify clusters with enriched gene transcription. Next, we focused on cells within these enriched clusters and calculated the prevalence of the target gene's transcription across different brain sections. This was done by normalizing the number of cells transcribing the gene in each section by the total number of cells in that section, expressed as a percentage. The results were plotted using a line plot to illustrate the gene's prevalence across brain sections. In panel F, to visualize the transcription of a specific gene in the top four brain sections, we implemented a function called "plot_4_best_sections" (in "Figures/Figure_2.ipynb"). This function aimed to identify and plot the sections with the highest gene transcription levels. For data preparation, we first prepared the dataset by selecting the relevant brain sections and ensuring that unassigned parcellation divisions were excluded. We merged this dataset with cluster membership information to provide context for the gene transcription data. For gene transcription calculation, the percentage of cells within each cluster transcribing the target gene above a defined threshold was calculated. This allowed us to identify clusters with enriched gene transcription. For section identification, we calculated the prevalence of the target gene's transcription in each brain section. Using these prevalence values, we identified the top four sections with the highest gene transcription. Peaks in the transcription data, spaced adequately

(C) Prevalence of Htr4 RNA across all classes of cells in scRNA-seq and MERFISH datasets. Inset represents the linear regression between the two datasets. On the right, absolute number of cells expressing Htr4 RNA in the scRNA-seq by class, ranked in descending order (top ten).

(D) Ranked prevalence of Htr4 RNA across divisions (left) and structures of enriched clusters found in the scRNA-seq dataset in the MERFISH dataset (right). Inset represents the proportion of cells expressing Htr4 RNA that belongs to enriched clusters.

(E) (Top) Prevalence of cells from enriched clusters across the anteroposterior axis, identified in the scRNA-seq dataset and cross-referenced in the MERFISH dataset. (Bottom) Average amount of RNA expression found in enriched clusters cross-referenced in the MERFISH dataset.

(F) Expression of Htr4 RNA detected by MERFISH in four representative slices. Border color represents the position on the anteroposterior axis.

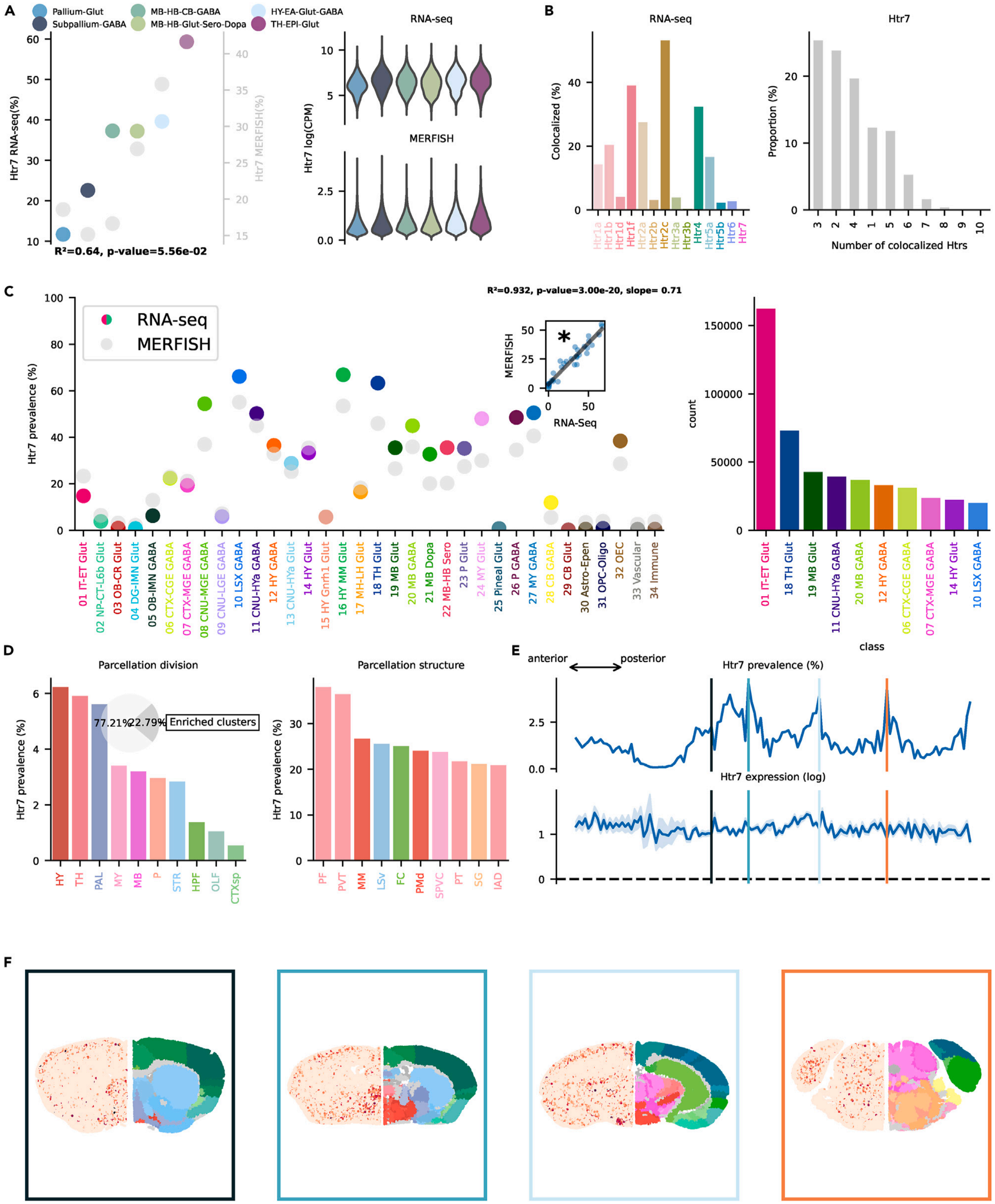

Figure 8. Htr7 transcription

(A) On the left, dotplot representing Htr7 prevalence across neighborhoods with squared Pearson correlation coefficient (R^2) between scRNA-seq and MERFISH datasets. On the right, violin plots representing the amount of Htr7 RNA detected using scRNA-seq (top) and MERFISH (bottom).

(B) Amount of co-localization with each Htr by cells expressing Htr7 RNA in the scRNA-seq dataset (left). Number of Htr RNA detected in cells expressing Htr7 RNA in the scRNA-seq dataset (right).

(legend continued on next page)

apart, were determined using the "find_peaks" function from scipy. The top four peaks were selected for visualization. For plotting, the gene transcription data were plotted for each of the top four sections. The "plot_slice" function was used to generate the plots for each section, and the border color of each subplot was set to match the assigned color for the respective section. The final figure comprised four subplots, each representing one of the top four brain sections with the highest gene transcription levels, providing a clear and comparative visualization of the gene transcription patterns across these key sections.

Data visualizer

The visualizer was built in Python using Matplotlib, Holoviews, and Panel libraries. It is available as a jupyter notebook ("Figures/Interactive_vizs.ipynb") and online (https://rdef654875678597657-5-ht-transcriptomics.hf.space). The jupyter notebook can be used locally by following the installation instructions available in https://github.com/RobertoDF/Transcriptomics-5-HT. The visualizer is deployed and accessible online on the Hugging Face portal. It is organized into four different dashboards: "Spatial MERFISH," "Gene by class/subclass/supertype/cluster," "Overview genes by class," and "Overview genes by brain structure." "Spatial MERFISH" and "Overview genes by brain structure" are associated with the MERFISH dataset, and remaining tabs are associated with the scRNA-seq dataset. Each dashboard's data source is annotated in the title. "Spatial MERFISH": five interactive controls enable the selections of different datasets from Zhang et al.,[12] namely brain section, gene, class, and subclass. The datasets available are two coronal (Zhuang-ABCA-1/2) and two sagittal (Zhuang-ABCA-3/4). The controls allow visualization of different slices, specific genes, and selected groups. The dashboard includes six panels: (1) line plot representing the proportion of cells selected across the spatial axis associated to each dataset, (2) line plot representing the amount of transcription across space of the selected gene, (3) line plot representing the percentage of cells across space in which RNA of the selected gene was detected (threshold set at 0.3), (4) barplot representing the percentage of Htr-positive cells in the selected slice grouped by brain structure (number in each bar is the absolute number of cells), and (5 and 6) slice selected with gene transcription (left) and atlas metadata (right). "Gene by class/subclass/supertype/cluster": this dashboard has two interactive controls for selecting neighborhood group and gene. For each class of neurons, three levels of visualization are provided: (1) violin plots, gene prevalence by subclass; (2) violin plots, prevalence by supertype; and (3) barplots, prevalence by cluster. "Overview genes by class": this dashboard includes four interactive controls for selecting class, subclass, type of grouping, and sorting. The plot can be grouped at different clustering depths: classes, subclasses, supertypes, and even individual clusters (the number of groups that can visualized at the same time is limited by the maximum recursion depth of Holoviews). The plot can be sorted by the group's alphabetical name or gene transcription. Gene prevalence is represented with a heatmap in which the color bar is updated according to the limits of the current selection. The y axis is populated by the name of the groups selected by the "Group by" selector. The x axis shows each Htr. "Overview genes by brain structure": this dashboard includes four interactive controls for selecting data source, division, neurotransmitter, and sorting. Gene prevalence is represented with a heatmap in which the color bar is updated according to the limits of the current selection. Gene prevalence is limited to cluster enriched in the according gene (prevalence within cluster of the gene >70%). The y axis is populated by the brain structures belonging to the currently selected brain division. For each division, we can refine our selection by isolating neurons releasing a specific neurotransmitter. The x axis shows each Htr. First, enriched clusters in the scRNA-seq dataset are identified, and the proportion of cells belonging to enriched clusters over the total number of cells per region is then analyzed. To handle cases where most cells do not belong to enriched clusters and are ignored, a "Data source selector" is used to bypass scRNA-seq data and MERFISH data used directly. In this case we look directly at the ratio of cells transcribing each gene over the total number of cells per region.

RESOURCE AVAILABILITY

Lead contact

Roberto De Filippo is the lead contact and can be reached at roberto.de-filippo@bccn-berlin.de.

Materials availability

This study did not generate new unique materials.

Data and code availability

The entire analysis is hosted on Github at https://github.com/RobertoDF/Transcriptomics-5-HT and https://doi.org/10.6084/m9.figshare.26325505.[56] For data analysis and visualization we employed mainly pandas, numpy, Matplotlib, scikit-learn, Panel, Holoviews, and Seaborn Python libraries. Within the "Figures" folder, "Figure_1.ipynb" and "Figure_2.ipynb" notebooks reproduce all figures contained in the paper. All parameters relative to the analysis are contained in Utils.Settings.py. Data are downloaded following the instructions provided by the Allen Institute (# point to your aws installation, see https://github.com/AllenInstitute/abc_atlas_access/blob/ecd803247b2c2dfa3ce2297880c9cfbf8c79cfab/notebooks/getting_started.ipynb); notebooks to download the scRNA-seq and MERFISH datasets are contained in the "Load_Data" folder. To explore the transcription of different genes, it is necessary to download the associated transcription matrices by changing the selected genes in the "Download_RNAseq_data.ipynb" notebook; this can be achieved by modifying the cells underneath the headings "Select genes scRNA-seq" and "Select genes MERFISH." It is also necessary to change the "family_name" and "genes_families" variables in Utils.Settings.py file.

ACKNOWLEDGMENTS

This study was supported by the German Research Foundation Deutsche Forschungsgemeinschaft, project 184695641 - SFB 958 and project 327654276 - SFB 1315; Germany's Excellence Strategy - Exc-2049-390688087; and the European Research Council under the European Union's Horizon 2020 research and innovation program (grant agreement no. 810580). We thank Willy Schiegel and Tiziano Zito for technical help with cluster computing. We thank members of the Schmitz lab for discussion.

AUTHOR CONTRIBUTIONS

Conceptualization, data curation, formal analysis, investigation, visualization, and supervision, R.D.F.; writing – original draft, R.D.F.; writing – review & editing, R.D.F.; funding acquisition and resources, D.S.

DECLARATION OF INTERESTS

The authors declare no competing interests.

SUPPLEMENTAL INFORMATION

Supplemental information can be found online at https://doi.org/10.1016/j.patter.2024.101048.

(C) Prevalence of Htr7 RNA across all classes of cells in scRNA-seq and MERFISH datasets. Inset represents the linear regression between the two datasets. On the right, absolute number of cells expressing Htr7 RNA in the scRNA-seq by class, ranked in descending order (top ten).
(D) Ranked prevalence of Htr7 RNA across divisions (left) and structures of enriched clusters found in the scRNA-seq dataset in the MERFISH dataset (right). Inset represents the proportion of cells expressing Htr7 RNA that belongs to enriched clusters.
(E) (Top) Prevalence of cells from enriched clusters across the anteroposterior axis, identified in the scRNA-seq dataset and cross-referenced in the MERFISH dataset. (Bottom) Average amount of RNA expression found in enriched clusters cross-referenced in the MERFISH dataset.
(F) Expression of Htr7 RNA detected by MERFISH in four representative slices. Border color represents the position on the anteroposterior axis.

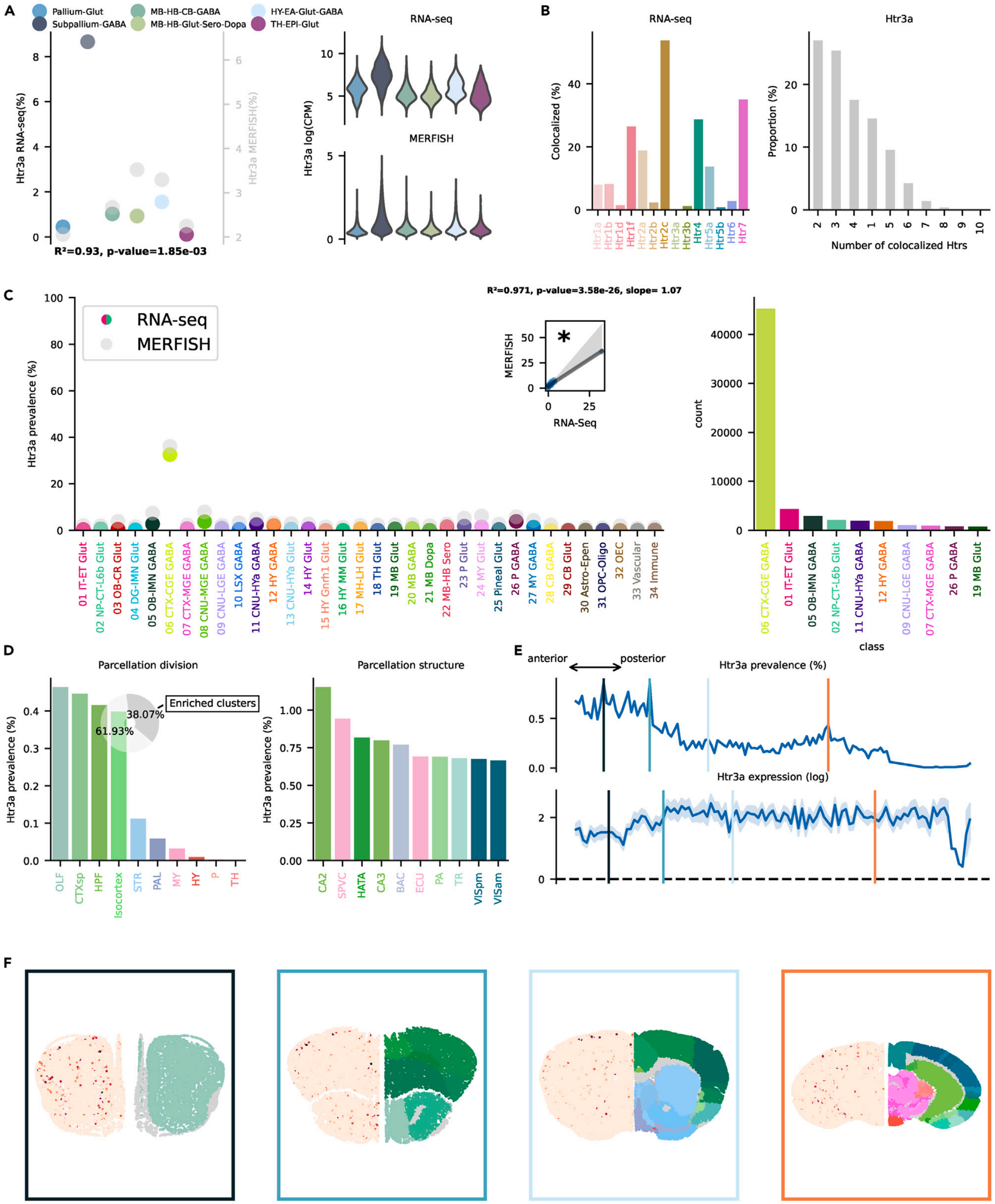

Figure 9. Htr3a transcription

(A) On the left, dotplot representing Htr3a prevalence across neighborhoods with squared Pearson correlation coefficient (R^2) between scRNA-seq and MERFISH datasets. On the right, violin plots representing the amount of Htr3a RNA detected using scRNA-seq (top) and MERFISH (bottom).

(B) Amount of co-localization with each Htr by cells expressing Htr3a RNA in the scRNA-seq dataset (left). Number of Htr RNA detected in cells expressing Htr3a RNA in the scRNA-seq dataset (right).

(legend continued on next page)

Received: April 8, 2024
Revised: June 19, 2024
Accepted: July 31, 2024
Published: August 29, 2024

REFERENCES

1. Dayan, P., and Huys, Q.J.M. (2009). Serotonin in Affective Control. Annu. Rev. Neurosci. *32*, 95–126.
2. Teixeira, C.M., Rosen, Z.B., Suri, D., Sun, Q., Hersh, M., Sargin, D., Dincheva, I., Morgan, A.A., Spivack, S., Krok, A.C., et al. (2018). Hippocampal 5-HT Input Regulates Memory Formation and Schaffer Collateral Excitation. Neuron *98*, 992–1004.e4.
3. Oikonomou, G., Altermatt, M., Zhang, R.W., Coughlin, G.M., Montz, C., Gradinaru, V., and Prober, D.A. (2019). The Serotonergic Raphe Promote Sleep in Zebrafish and Mice. Neuron *103*, 686–701.e8.
4. Lee, M.D., and Clifton, P.G. (2010). CHAPTER 3.3 - Role of the Serotonergic System in Appetite and Ingestion Control. In Handbook of Behavioral Neuroscience, C.P. Müller and B.L. Jacobs, eds. (Elsevier), pp. 331–345. https://doi.org/10.1016/S1569-7339(10)70088-6.
5. Homberg, J.R. (2012). Serotonin and decision making processes. Neurosci. Biobehav. Rev. *36*, 218–236.
6. Descarries, L., Riad, M., and Parent, M. (2010). CHAPTER 1.4 - Ultrastructure of the Serotonin Innervation in the Mammalian Central Nervous System. In Handbook of Behavioral Neuroscience, C.P. Müller and B.L. Jacobs, eds. (Elsevier), pp. 65–101. https://doi.org/10.1016/S1569-7339(10)70072-2.
7. Marin, P., Bécamel, C., Chaumont-Dubel, S., Vandermoere, F., Bockaert, J., and Claeysen, S. (2020). Chapter 5 - Classification and signaling characteristics of 5-HT receptors: toward the concept of 5-HT receptosomes. In Handbook of Behavioral Neuroscience, C.P. Müller and K.A. Cunningham, eds. (Elsevier), pp. 91–120. https://doi.org/10.1016/B978-0-444-64125-0.00005-0.
8. Vilaró, M.T., Cortés, R., Mengod, G., and Hoyer, D. (2020). Chapter 6 - Distribution of 5-HT receptors in the central nervous system: an update. In Handbook of Behavioral Neuroscience, C.P. Müller and K.A. Cunningham, eds. (Elsevier), pp. 121–146. https://doi.org/10.1016/B978-0-444-64125-0.00006-2.
9. Hawrylycz, M.J., Lein, E.S., Guillozet-Bongaarts, A.L., Shen, E.H., Ng, L., Miller, J.A., van de Lagemaat, L.N., Smith, K.A., Ebbert, A., Riley, Z.L., et al. (2012). An anatomically comprehensive atlas of the adult human brain transcriptome. Nature *489*, 391–399.
10. Salvan, P., Fonseca, M., Winkler, A.M., Beauchamp, A., Lerch, J.P., and Johansen-Berg, H. (2023). Serotonin regulation of behavior via large-scale neuromodulation of serotonin receptor networks. Nat. Neurosci. *26*, 53–63.
11. Yao, Z., van Velthoven, C.T.J., Kunst, M., Zhang, M., McMillen, D., Lee, C., Jung, W., Goldy, J., Abdelhak, A., Aitken, M., et al. (2023). A high-resolution transcriptomic and spatial atlas of cell types in the whole mouse brain. Nature *624*, 317–332.
12. Zhang, M., Pan, X., Jung, W., Halpern, A.R., Eichhorn, S.W., Lei, Z., Cohen, L., Smith, K.A., Tasic, B., Yao, Z., et al. (2023). Molecularly defined and spatially resolved cell atlas of the whole mouse brain. Nature *624*, 343–354.
13. Lundberg, S., and Lee, S.-I. (2017). A Unified Approach to Interpreting Model Predictions. Preprint at arXiv *1234*, 1234. https://doi.org/10.48550/arXiv.1705.07874.
14. Lundberg, S.M., Erion, G., Chen, H., DeGrave, A., Prutkin, J.M., Nair, B., Katz, R., Himmelfarb, J., Bansal, N., and Lee, S.-I. (2020). From local explanations to global understanding with explainable AI for trees. Nat. Mach. Intell. *2*, 56–67.
15. Tanaka, K.F., Samuels, B.A., and Hen, R. (2012). Serotonin receptor expression along the dorsal-ventral axis of mouse hippocampus. Philos. Trans. R. Soc. Lond. B Biol. Sci. *367*, 2395–2401.
16. Sharp, T., and Barnes, N.M. (2020). Central 5-HT receptors and their function; present and future. Neuropharmacology *177*, 108155.
17. McCorvy, J.D., and Roth, B.L. (2015). Structure and function of serotonin G protein-coupled receptors. Pharmacol. Ther. *150*, 129–142.
18. Parks, C.L., Robinson, P.S., Sibille, E., Shenk, T., and Toth, M. (1998). Increased anxiety of mice lacking the serotonin 1A receptor. Proc. Natl. Acad. Sci. USA *95*, 10734–10739.
19. Harmer, C.J., Duman, R.S., and Cowen, P.J. (2017). How do antidepressants work? New perspectives for refining future treatment approaches. Lancet Psychiatr. *4*, 409–418.
20. Yohn, C.N., Gergues, M.M., and Samuels, B.A. (2017). The role of 5-HT receptors in depression. Mol. Brain *10*, 28.
21. Cowen, P.J., and Browning, M. (2015). What has serotonin to do with depression? World Psychiatr. *14*, 158–160.
22. Moncrieff, J., Cooper, R.E., Stockmann, T., Amendola, S., Hengartner, M.P., and Horowitz, M.A. (2023). The serotonin theory of depression: a systematic umbrella review of the evidence. Mol. Psychiatr. *28*, 3243–3256.
23. Goadsby, P.J. (2007). Serotonin receptor ligands: treatments of acute migraine and cluster headache. Handb. Exp. Pharmacol. 129–143.
24. Conn, K., Milton, L.K., Huang, K., Munguba, H., Ruuska, J., Lemus, M.B., Greaves, E., Homman-Ludiye, J., Oldfield, B.J., and Foldi, C.J. (2024). Psilocybin restrains activity-based anorexia in female rats by enhancing cognitive flexibility: contributions from 5-HT1A and 5-HT2A receptor mechanisms. Mol. Psychiatr. 1–14.
25. Winson, J. (1978). Loss of hippocampal theta rhythm results in spatial memory deficit in the rat. Science (New York, NY) *201*, 160–163.
26. Haj-Dahmane, S., Hamon, M., and Lanfumey, L. (1991). K+ channel and 5-hydroxytryptamine1A autoreceptor interactions in the rat dorsal raphe nucleus: Anin vitro electrophysiological study. Neuroscience *41*, 495–505.
27. Sprouse, J.S., and Aghajanian, G.K. (1987). Electrophysiological responses of serotoninergic dorsal raphe neurons to 5-HT1A and 5-HT1B agonists. Synapse *1*, 3–9.
28. Schmitz, D., Empson, R.M., Gloveli, T., and Heinemann, U. (1995). Serotonin reduces synaptic excitation of principal cells in the superficial layers of rat hippocampal-entorhinal cortex combined slices. Neurosci. Lett. *190*, 37–40.
29. de Filippo, R., Rost, B.R., Stumpf, A., Cooper, C., Tukker, J.J., Harms, C., Beed, P., and Schmitz, D. (2021). Somatostatin interneurons activated by 5-HT(2A) receptor suppress slow oscillations in medial entorhinal cortex. eLife *10*, e66960. https://doi.org/10.7554/eLife.66960.
30. Kia, H.K., Brisorgueil, M.J., Daval, G., Langlois, X., Hamon, M., and Vergé, D. (1996). Serotonin1A receptors are expressed by a subpopulation of cholinergic neurons in the rat medial septum and diagonal band of Broca—a double immunocytochemical study. Neuroscience *74*, 143–154.
31. Wu, X., Morishita, W., Beier, K.T., Heifets, B.D., and Malenka, R.C. (2021). 5-HT modulation of a medial septal circuit tunes social memory stability. Nature *599*, 96–101.

(C) Prevalence of Htr3a RNA across all classes of cells in scRNA-seq and MERFISH datasets. Inset represents the linear regression between the two datasets. On the right, absolute number of cells expressing Htr3a RNA in the scRNA-seq by class, ranked in descending order (top ten).
(D) Ranked prevalence of Htr3a RNA across divisions (left) and structures of enriched clusters found in the scRNA-seq dataset in the MERFISH dataset (right). Inset represents the proportion of cells expressing Htr3a RNA that belongs to enriched clusters.
(E) (Top) Prevalence of cells from enriched clusters across the anteroposterior axis, identified in the scRNA-seq dataset and cross-referenced in the MERFISH dataset. (Bottom) Average amount of RNA expression found in enriched clusters cross-referenced in the MERFISH dataset.
(F) Expression of Htr3a RNA detected by MERFISH in four representative slices. Border color represents the position on the anteroposterior axis.

32. Maroteaux, L., Saudou, F., Amlaiky, N., Boschert, U., Plassat, J.L., and Hen, R. (1992). Mouse 5HT1B serotonin receptor: cloning, functional expression, and localization in motor control centers. Proc. Natl. Acad. Sci. USA *89*, 3020–3024.
33. Pommer, S., Akamine, Y., Schiffmann, S.N., de Kerchove d'Exaerde, A., and Wickens, J.R. (2021). The Effect of Serotonin Receptor 5-HT1B on Lateral Inhibition between Spiny Projection Neurons in the Mouse Striatum. J. Neurosci. *41*, 7831–7847.
34. Hjorth, S., and Tao, R. (1991). The putative 5-HT1B receptor agonist CP-93,129 suppresses rat hippocampal 5-HT release in vivo: comparison with RU 24969. Eur. J. Pharmacol. *209*, 249–252.
35. Bruinvels, A.T., Landwehrmeyer, B., Gustafson, E.L., Durkin, M.M., Mengod, G., Branchek, T.A., Hoyer, D., and Palacios, J.M. (1994). Localization of 5-HT1B, 5-HT1Dα, 5-HT1E and 5-HT1F receptor messenger RNA in rodent and primate brain. Neuropharmacology *33*, 367–386.
36. Vila-Pueyo, M. (2018). Targeted 5-HT(1F) Therapies for Migraine. Neurotherapeutics *15*, 291–303.
37. Chou, M.-Y., Amo, R., Kinoshita, M., Cherng, B.-W., Shimazaki, H., Agetsuma, M., Shiraki, T., Aoki, T., Takahoko, M., Yamazaki, M., et al. (2016). Social conflict resolution regulated by two dorsal habenular subregions in zebrafish. Science (New York, NY) *352*, 87–90.
38. Soria-Gomez, E., Busquets-Garcia, A., Hu, F., Mehidi, A., Cannich, A., Roux, L., Louit, I., Alonso, L., Wiesner, T., and Georges, F. (2015). Habenular CB1 receptors control the expression of aversive memories. Neuron *88*, 306–313.
39. Yamaguchi, T., Danjo, T., Pastan, I., Hikida, T., and Nakanishi, S. (2013). Distinct roles of segregated transmission of the septo-habenular pathway in anxiety and fear. Neuron *78*, 537–544.
40. He, Y., Brouwers, B., Liu, H., Liu, H., Lawler, K., Mendes de Oliveira, E., Lee, D.-K., Yang, Y., Cox, A.R., Keogh, J.M., et al. (2022). Human loss-of-function variants in the serotonin 2C receptor associated with obesity and maladaptive behavior. Nat. Med. *28*, 2537–2546.
41. Yao, T., He, J., Cui, Z., Wang, R., Bao, K., Huang, Y., Wang, R., and Liu, T. (2021). Central 5-HTR2C in the Control of Metabolic Homeostasis. Front. Endocrinol. *12*, 694204.
42. Nichols, D.E. (2016). Psychedelics. Pharmacol. Rev. *68*, 264–355.
43. Morici, J.F., Miranda, M., Gallo, F.T., Zanoni, B., Bekinschtein, P., and Weisstaub, N.V. (2018). 5-HT2a receptor in mPFC influences context-guided reconsolidation of object memory in perirhinal cortex. eLife *7*, e33746.
44. de Quervain, D.J.F., Henke, K., Aerni, A., Coluccia, D., Wollmer, M.A., Hock, C., Nitsch, R.M., and Papassotiropoulos, A. (2003). A functional genetic variation of the 5-HT2a receptor affects human memory. Nat. Neurosci. *6*, 1141–1142.
45. De Filippo, R., and Schmitz, D. (2024). Synthetic Surprise as the Foundation of the Psychedelic Experience. Neurosci. Biobehav. Rev. *157*, 105538. https://doi.org/10.1016/j.neubiorev.2024.105538.
46. Huang, W.-C., Peng, Z., Murdock, M.H., Liu, L., Mathys, H., Davila-Velderrain, J., Jiang, X., Chen, M., Ng, A.P., Kim, T., et al. (2023). Lateral mammillary body neurons in mouse brain are disproportionately vulnerable in Alzheimer's disease. Sci. Transl. Med. *15*, eabq1019.
47. Roy, D.S., Kitamura, T., Okuyama, T., Ogawa, S.K., Sun, C., Obata, Y., Yoshiki, A., and Tonegawa, S. (2017). Distinct Neural Circuits for the Formation and Retrieval of Episodic Memories. Cell *170*, 1000–1012.e19.
48. Doss, M.K., Madden, M.B., Gaddis, A., Nebel, M.B., Griffiths, R.R., Mathur, B.N., and Barrett, F.S. (2022). Models of psychedelic drug action: modulation of cortical-subcortical circuits. Brain *145*, 441–456.
49. Lebow, M.A., and Chen, A. (2016). Overshadowed by the amygdala: the bed nucleus of the stria terminalis emerges as key to psychiatric disorders. Mol. Psychiatr. *21*, 450–463.
50. López-Giménez, J.F., Vilaró, M.T., Palacios, J.M., and Mengod, G. (2001). Mapping of 5-HT2A receptors and their mRNA in monkey brain: [3H] MDL100,907 autoradiography and in situ hybridization studies. J. Comp. Neurol. *429*, 571–589.
51. Humby, T., Patel, Y., Carter, J., Stokes, L.J.G., Rogers, R.D., and Wilkinson, L.S. (2019). Feeding behaviour, risk-sensitivity and response control: effects of 5-HT(2C) receptor manipulations. Philos. Trans. R. Soc. Lond. B Biol. Sci. *374*, 20180144.
52. Karayol, R., Medrihan, L., Warner-Schmidt, J.L., Fait, B.W., Rao, M.N., Holzner, E.B., Greengard, P., Heintz, N., and Schmidt, E.F. (2021). Serotonin receptor 4 in the hippocampus modulates mood and anxiety. Mol. Psychiatr. *26*, 2334–2349.
53. Maricq, A.V., Peterson, A.S., Brake, A.J., Myers, R.M., and Julius, D. (1991). Primary structure and functional expression of the 5HT3 receptor, a serotonin-gated ion channel. Science (New York, NY) *254*, 432–437.
54. Walstab, J., Rappold, G., and Niesler, B. (2010). 5-HT3 receptors: role in disease and target of drugs. Pharmacol. Ther. *128*, 146–169.
55. Davies, P.A., Pistis, M., Hanna, M.C., Peters, J.A., Lambert, J.J., Hales, T.G., and Kirkness, E.F. (1999). The 5-HT3B subunit is a major determinant of serotonin-receptor function. Nature *397*, 359–363.
56. De Filippo, R. (2024). Transcriptomic Mapping of the 5-HT Receptor Landscape (Version 1). https://doi.org/10.6084/m9.figshare.26325505.v1.

Patterns, Volume *5*

Supplemental information

Transcriptomic mapping of the 5-HT receptor landscape

Roberto De Filippo and Dietmar Schmitz

Transcriptomic Mapping of the 5-HT Receptor Landscape

Roberto De Filippo[1] and Dietmar Schmitz[12345]

[1] Charité Universitätsmedizin Berlin, corporate member of Freie Universität Berlin, Humboldt-Universität zu Berlin, and Berlin Institute of Health; Neuroscience Research Center, 10117 Berlin, Germany.
[2] German Center for Neurodegenerative Diseases (DZNE) Berlin, 10117 Berlin, Germany.
[3] Charité-Universitätsmedizin Berlin, corporate member of Freie Universität Berlin, Humboldt-Universität Berlin, and Berlin Institute of Health, Einstein Center for Neuroscience, 10117 Berlin, Germany.
[4] Charité-Universitätsmedizin Berlin, corporate member of Freie Universität Berlin, Humboldt-Universität Berlin, and Berlin Institute of Health, NeuroCure Cluster of Excellence, 10117 Berlin, Germany.
[5] Humboldt-Universität zu Berlin, Bernstein Center for Computational Neuroscience, Philippstr. 13, 10115 Berlin, Germany.

* Corresponding author and lead contact: roberto.de-filippo@bccn-berlin.de

Supplemental Information

Supplementary Figures

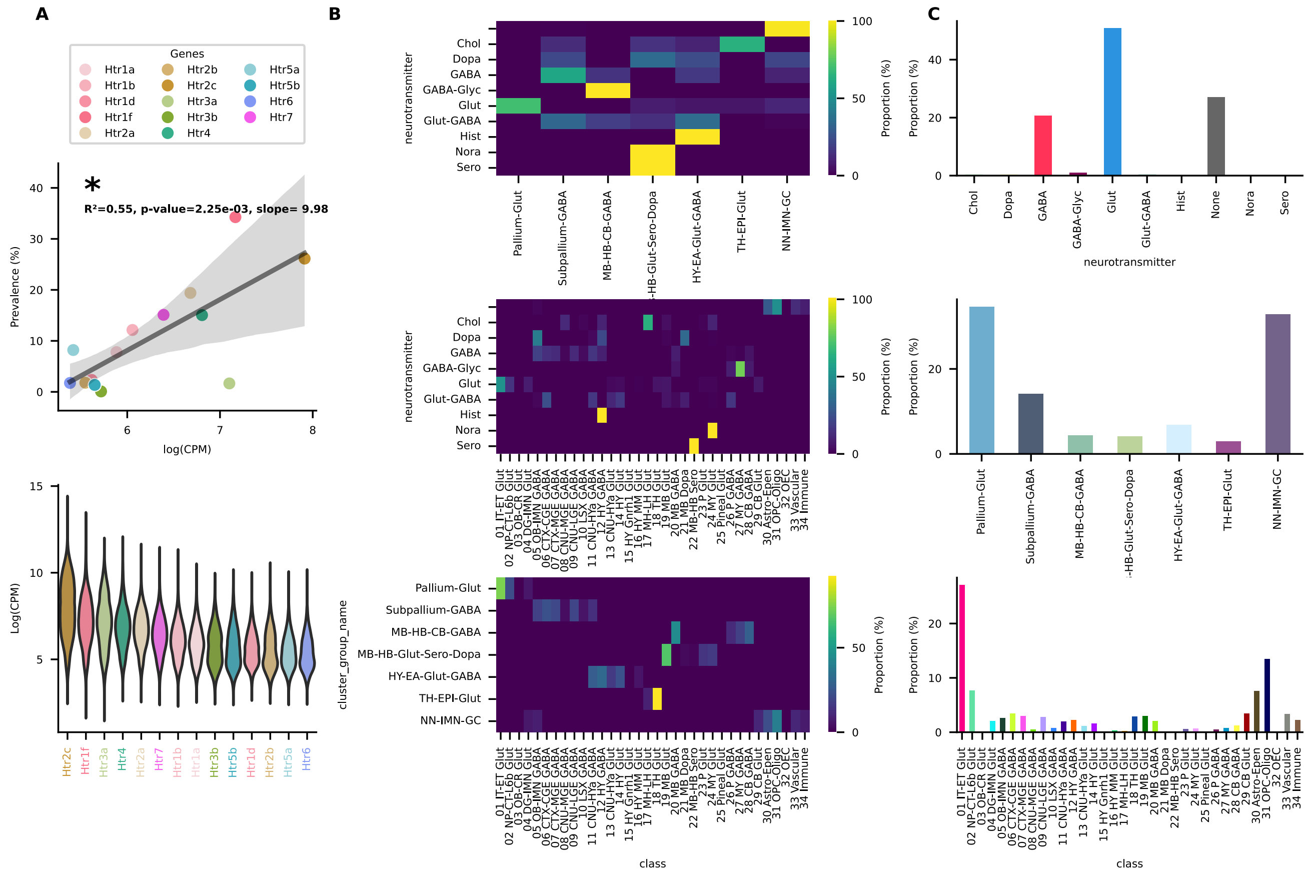

Figure S1. Htrs transcription and hierarchical clustering visualization.

(A) Top: Linear regression model fit between prevalence (y axis) and amount of intracellular expression (x axis) for each Htrs. Bottom: violin plots showing distribution of intracellular amount of expression for each Htrs. (B) Heatmaps showing relationship between neurotransmitter, class and neighborhoods. (C) Bar plots representing the size of each group when cells are categorized by neurotransmitter (top), neighborhood (middle) and class (botttom).

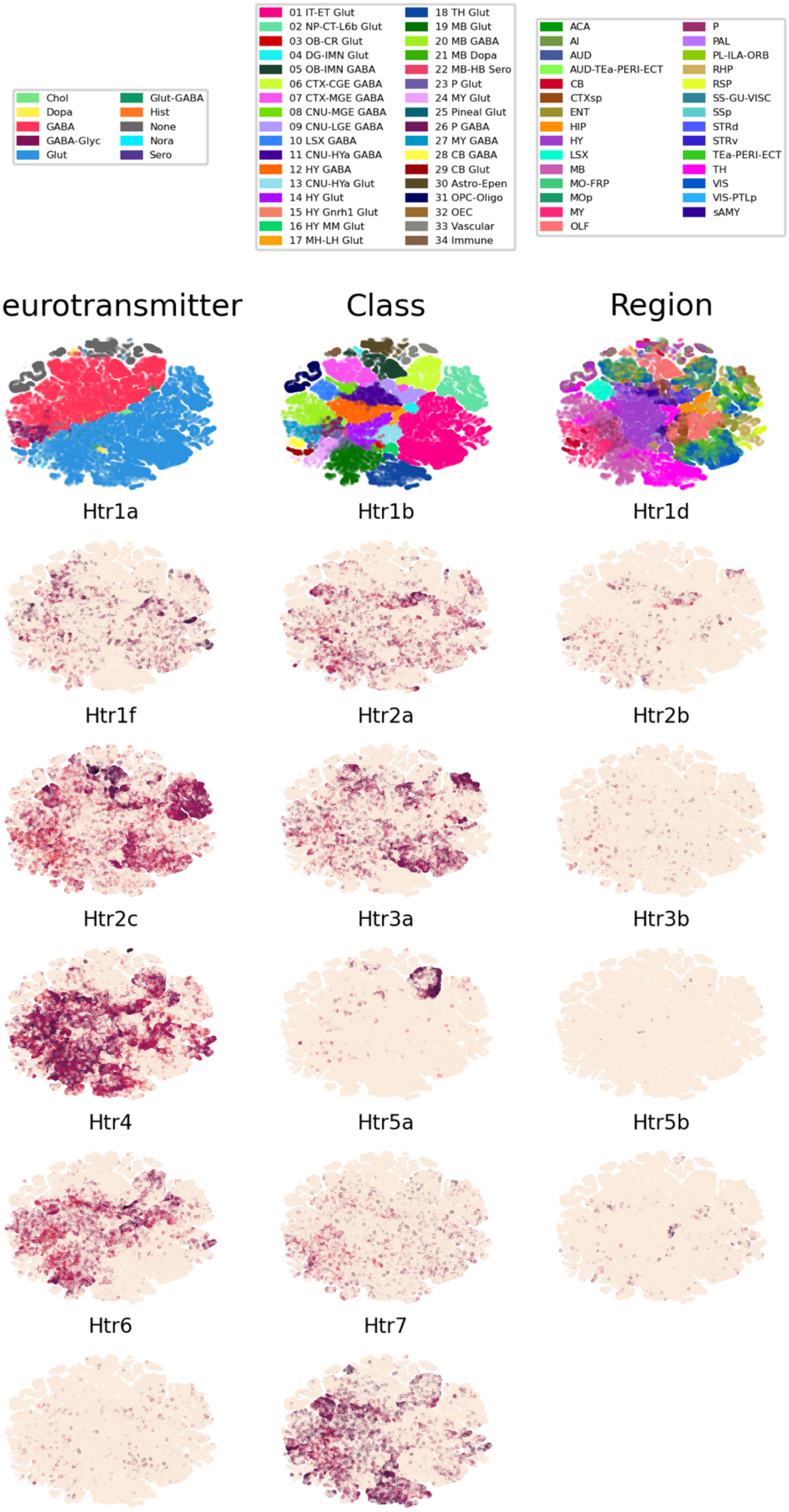

Figure S2. UMAP distribution of each Htr.

UMAP-associated neurotransmitter, class and dissection region metadata (first row) and UMAP distribution of each Htr.

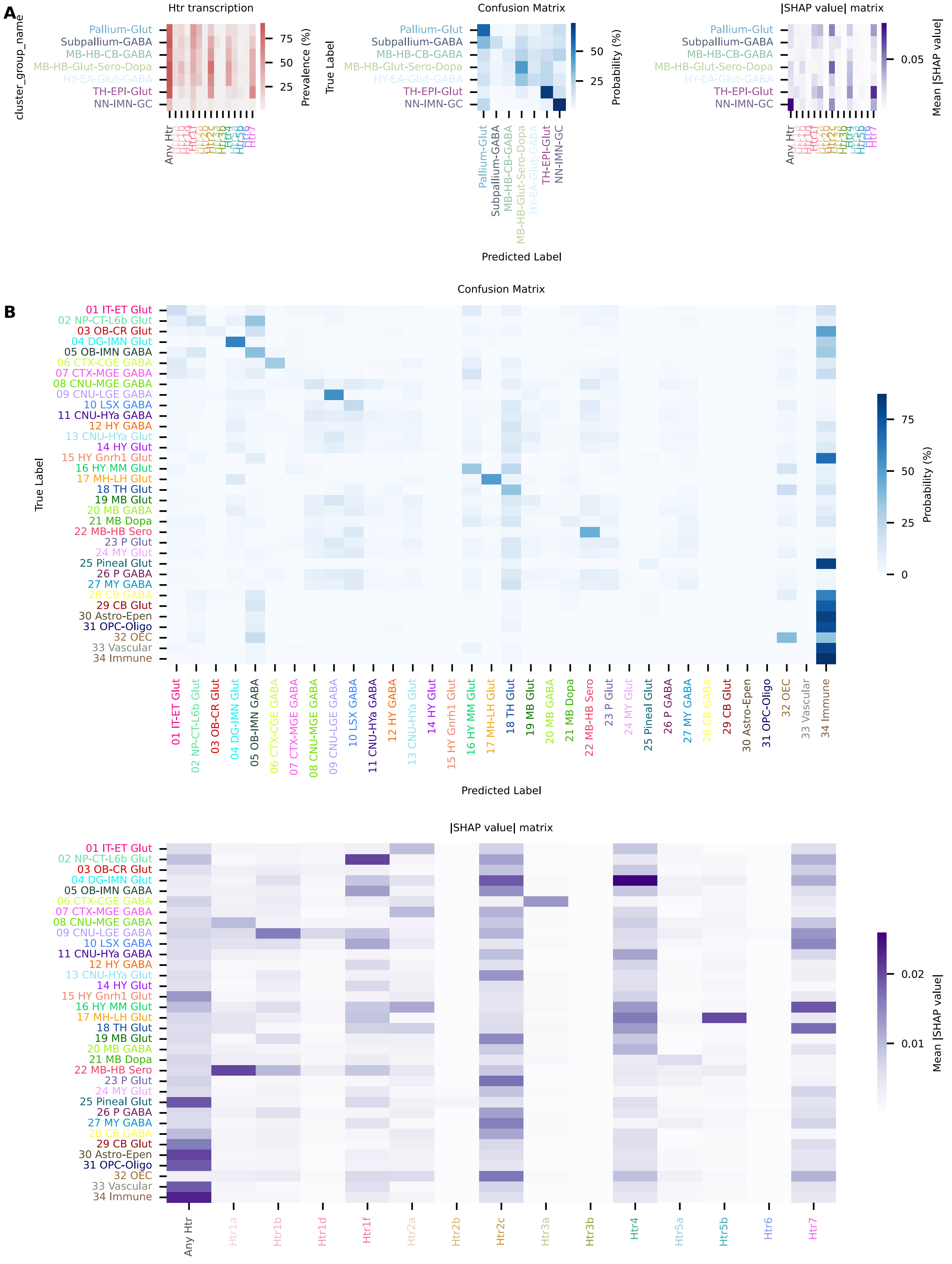

Figure S3. Htrs features across neighborhoods and classes.

(A) Htrs prevalence across neighborhoods (left). Confusion matrix related to the decoding of neighboroods classification from Htrs transcription (middle). SHAP values matrix associated to the neighborhood decoding (right). (B) Confusion matrix related to the decoding of classes

classification from Htrs transcription (left). SHAP values matrix associated to the class decoding (right).

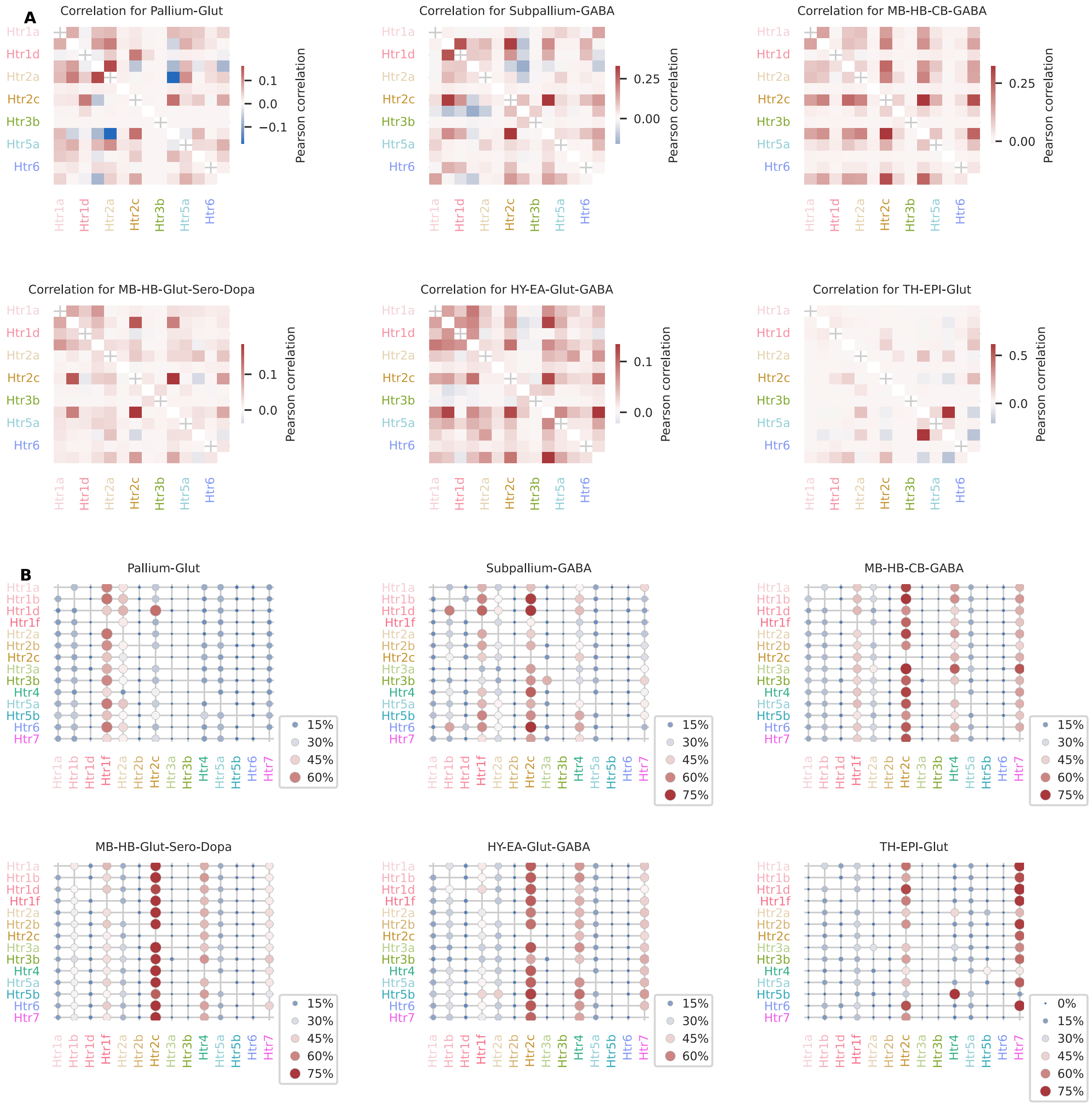

Figure S4. Correlation and co-localization patterns across neighborhoods.

(A) Correlation matrices between pairs of Htrs in each neighborhood. (B) co-localization matrices of Htrs transcription in each neighborhood.

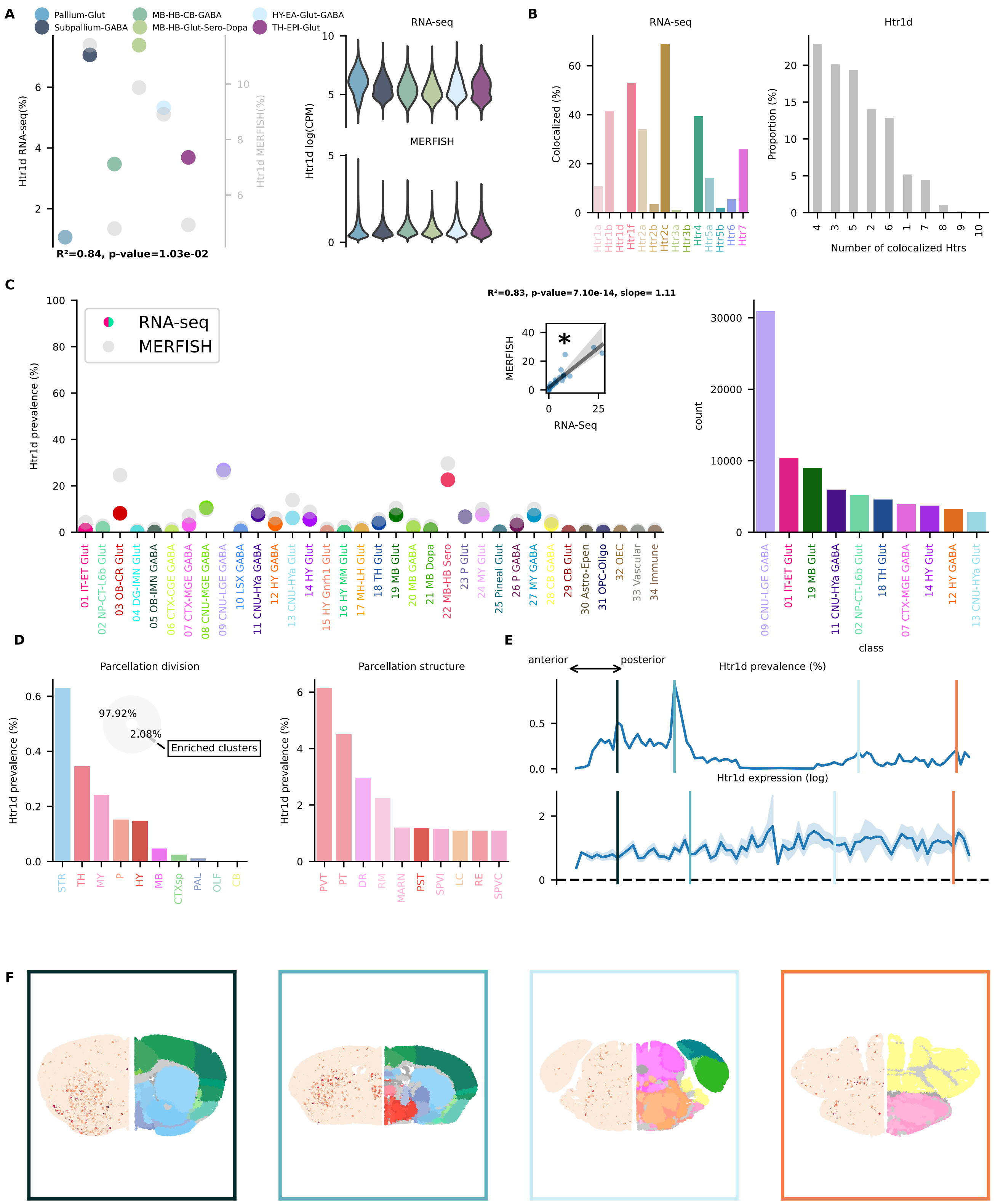

Figure S5. Htr1d transcription.

(A) On the left, Htr1d prevalence across neighborhoods with squared Pearson correlation coefficient (R^2) between scRNA-seq and MERFISH dataset. On the right, amount of Htr1d RNA detected using scRNA-seq (top) and MERFISH (bottom). (B) Amount of co-localization with each Htrs by cells expressing Htr1d RNA (left). Number of Htrs RNA detected in cells expressing Htr1d RNA (right). (C) Prevalence of Htr1d RNA across all classes of cells in

scRNA-seq and MERFISH dataset. Inset represents the linear regression between the two datasets. On the right, absolute number of cells expressing Htr1d RNA by class ranked in descending order (top ten). (D) Prevalence of Htr1d RNA across divisions (left) and structures (right). Inset represents the proportion of cells expressing Htr1d RNA that belongs to enriched clusters. (E) Top: Prevalence of cells from enriched clusters across the antero-posterior axis, identified in the scscRNA-seq dataset and cross-referenced in the MERFISH dataset. Bottom: average amount of RNA expression found in enriched clusters cross-referenced in the MERFISH dataset. (F) Expression of Htr1d RNA detected by MERFISH in 4 representative slices. Border color represents the position on the antero-posterior axis.

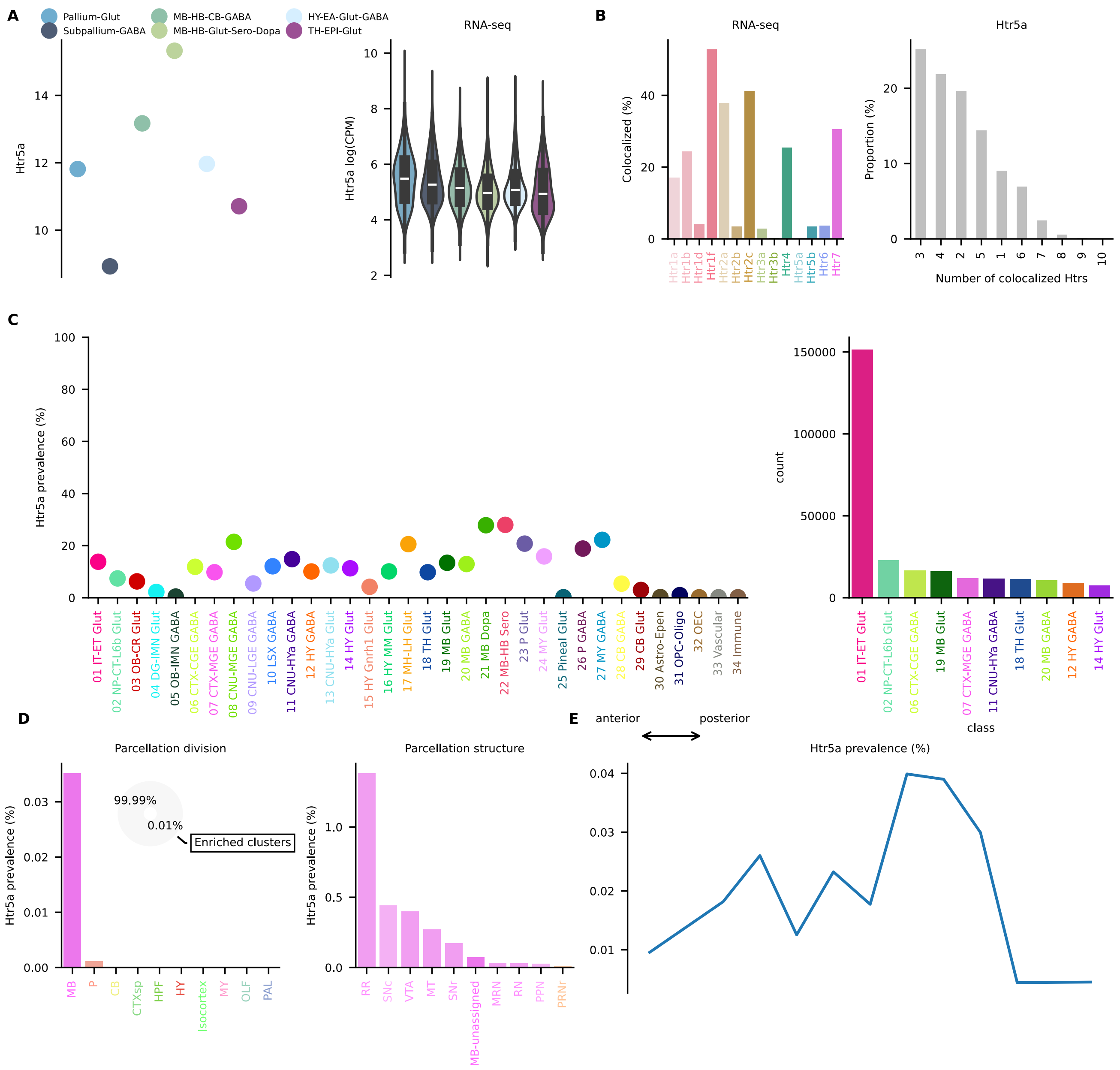

Supplementary Figure S6. Htr5a transcription.

(A) On the left, Htr5a prevalence across neighborhoods with squared Pearson correlation coefficient (R^2) between scRNA-seq and MERFISH dataset. On the right, amount of Htr5a RNA detected using scRNA-seq (top) and MERFISH (bottom). (B) Amount of co-localization with each Htrs by cells expressing Htr5a RNA (left). Number of Htrs RNA detected in cells expressing Htr5a RNA (right). (C) Prevalence of Htr5a RNA across all classes of cells in scRNA-seq and MERFISH dataset. Inset represents the linear regression between the two datasets. On the right, absolute number of cells expressing Htr5a RNA by class ranked in descending order (top ten). (D) Prevalence of Htr5a RNA across divisions (left) and structures (right). Inset represents the proportion of cells expressing Htr5a RNA that belongs to enriched clusters. (E)

Prevalence of cells from enriched clusters across the antero-posterior axis, identified in the scscRNA-seq dataset and cross-referenced in the MERFISH dataset.

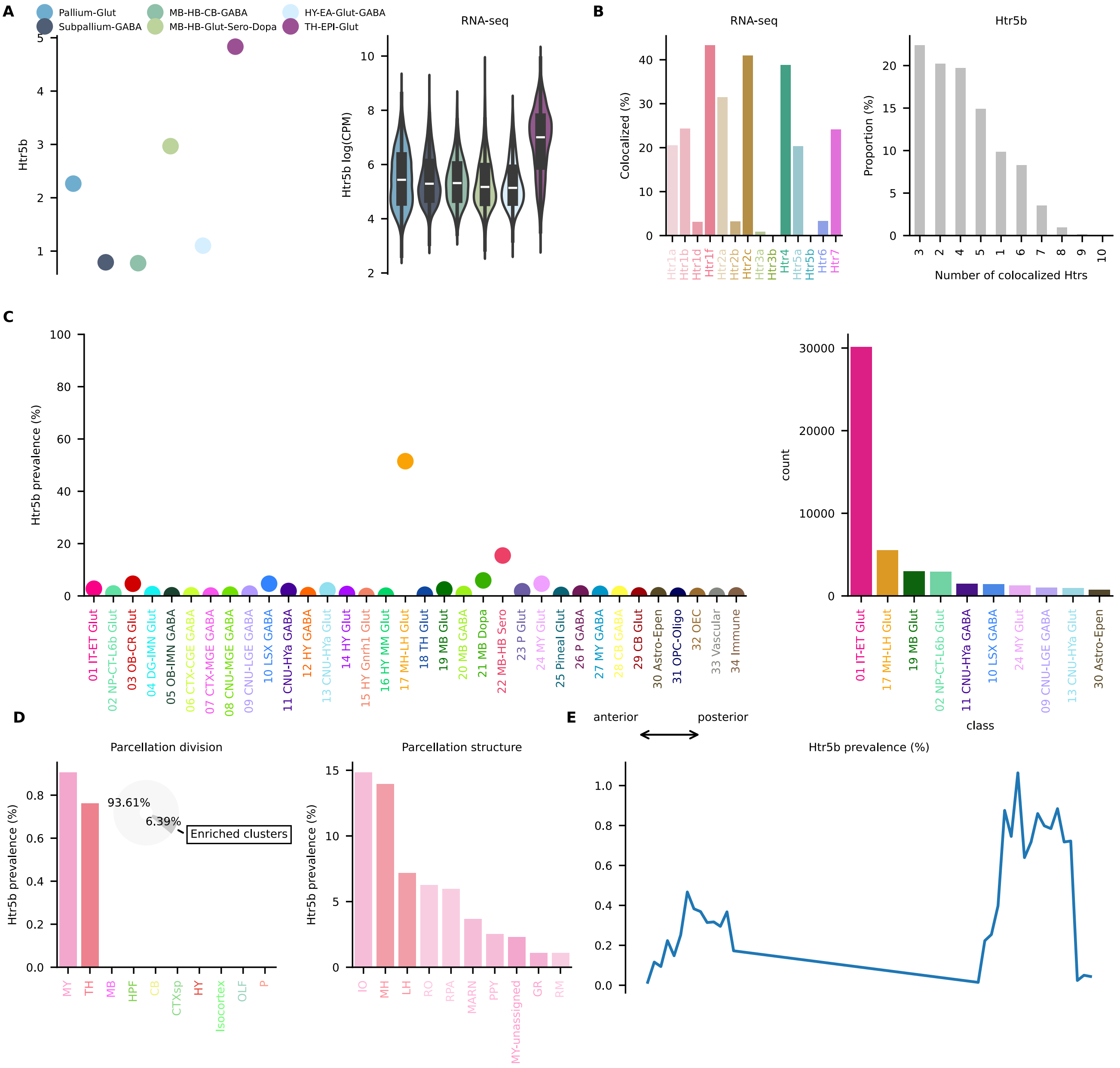

Figure S7. Htr5b transcription.

(A) On the left, Htr5b prevalence across neighborhoods with squared Pearson correlation coefficient (R^2) between scRNA-seq and MERFISH dataset. On the right, amount of Htr5b RNA detected using scRNA-seq (top) and MERFISH (bottom). (B) Amount of co-localization with each Htrs by cells expressing Htr5b RNA (left). Number of Htrs RNA detected in cells expressing Htr5b RNA (right). (C) Prevalence of Htr5b RNA across all classes of cells in scRNA-seq and MERFISH dataset. Inset represents the linear regression between the two datasets. On the right, absolute number of cells expressing Htr5b RNA by class ranked in descending order (top ten). (D) Prevalence of Htr5b RNA across divisions (left) and structures (right). Inset represents the proportion of cells expressing Htr5b RNA that belongs to enriched

clusters. (E) Prevalence of cells from enriched clusters across the antero-posterior axis, identified in the scscRNA-seq dataset and cross-referenced in the MERFISH dataset.

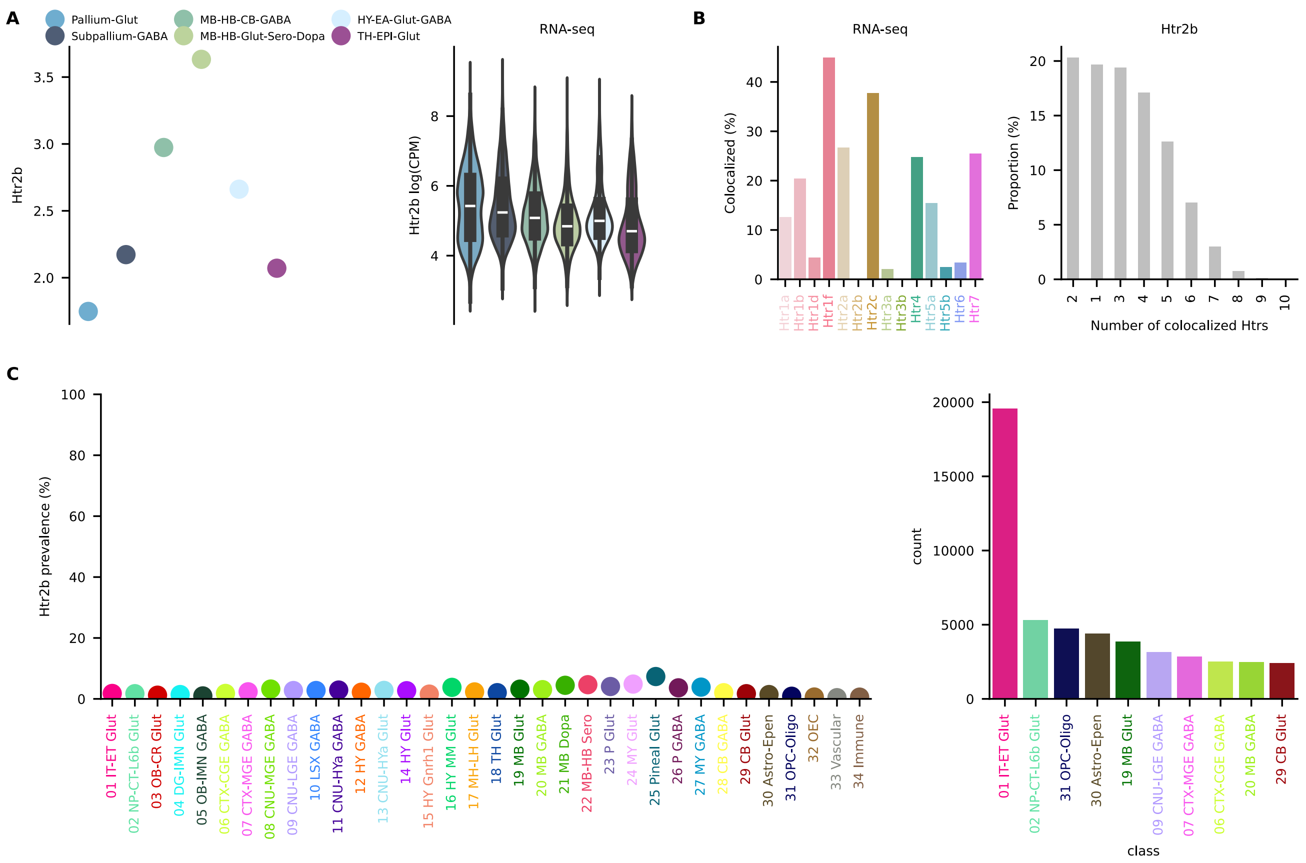

Supplementary Figure S8. Htr2b transcription.

(A) On the left, Htr2b prevalence across neighborhoods with squared Pearson correlation coefficient (R^2) between scRNA-seq and MERFISH dataset. On the right, amount of Htr2b RNA detected using scRNA-seq (top) and MERFISH (bottom). (B) Amount of co-localization with each Htrs by cells expressing Htr2b RNA (left). Number of Htrs RNA detected in cells expressing Htr2b RNA (right). (C) Prevalence of Htr2b RNA across all classes of cells in scRNA-seq and MERFISH dataset. Inset represents the linear regression between the two datasets. On the right, absolute number of cells expressing Htr2b RNA by class ranked in descending order (top ten).

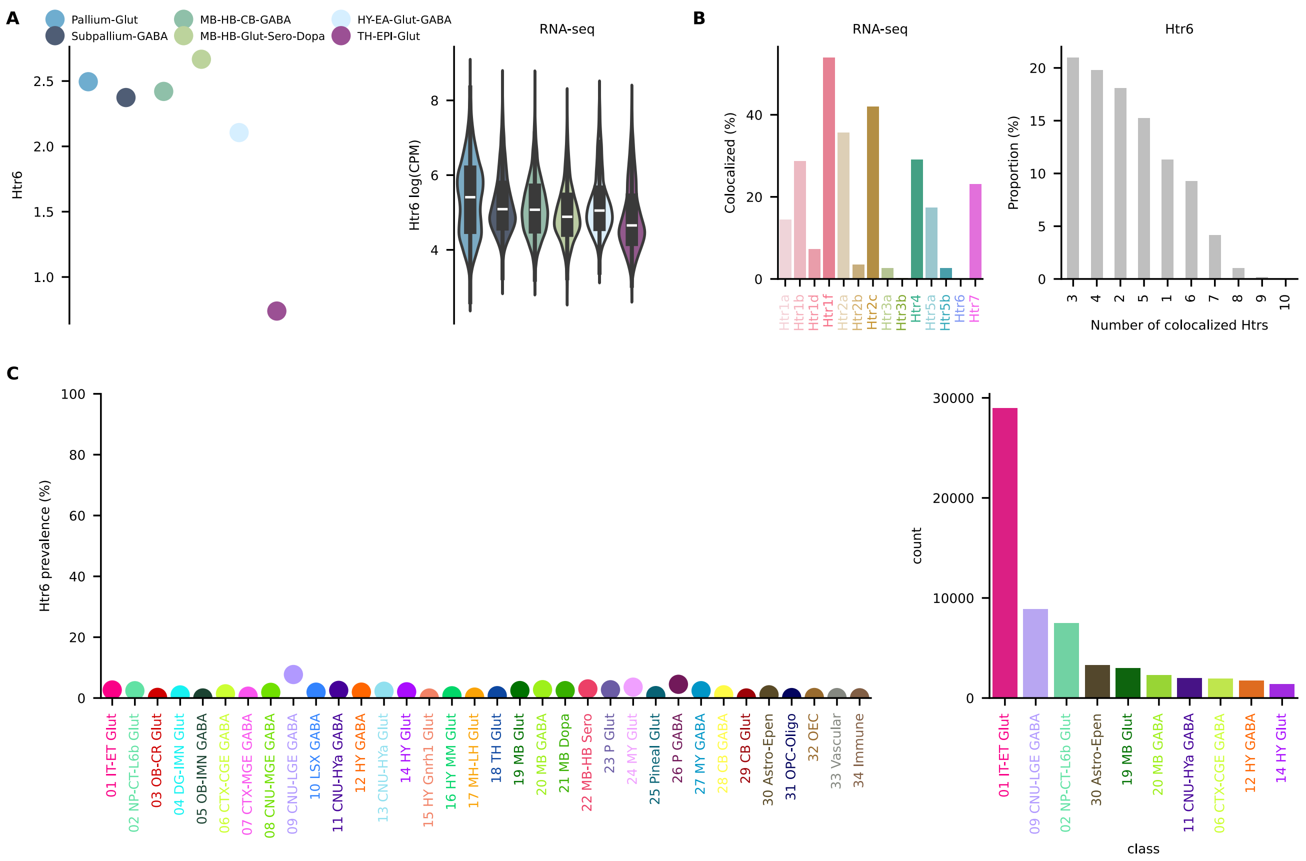

Figure S9. Htr6 transcription.

(A) On the left, Htr6 prevalence across neighborhoods with squared Pearson correlation coefficient (R^2) between scRNA-seq and MERFISH dataset. On the right, amount of Htr6 RNA detected using scRNA-seq (top) and MERFISH (bottom). (B) Amount of co-localization with each Htrs by cells expressing Htr6 RNA (left). Number of Htrs RNA detected in cells expressing Htr6 RNA (right). (C) Prevalence of Htr6 RNA across all classes of cells in scRNA-seq and MERFISH dataset. Inset represents the linear regression between the two datasets. On the right, absolute number of cells expressing Htr6 RNA by class ranked in descending order (top ten).

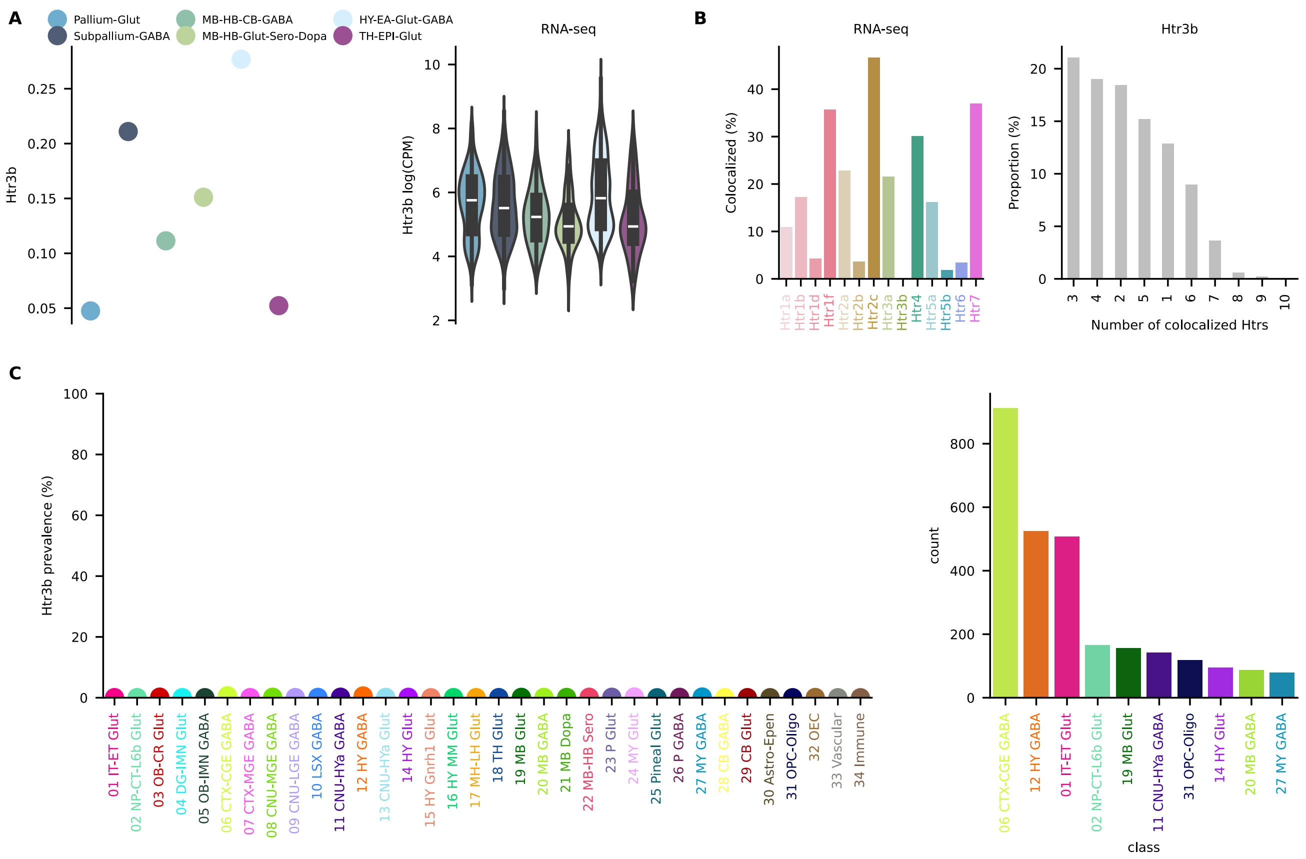

Figure S10. Htr3b transcription.

(A) On the left, Htr3b prevalence across neighborhoods with squared Pearson correlation coefficient (R^2) between scRNA-seq and MERFISH dataset. On the right, amount of Htr3b RNA detected using scRNA-seq (top) and MERFISH (bottom). (B) Amount of co-localization with each Htrs by cells expressing Htr3b RNA (left). Number of Htrs RNA detected in cells expressing Htr3b RNA (right). (C) Prevalence of Htr3b RNA across all classes of cells in scRNA-seq and MERFISH dataset. Inset represents the linear regression between the two datasets. On the right, absolute number of cells expressing Htr3b RNA by class ranked in descending order (top ten).

name	description
**HY-EA-Glut-GABA**	Glutamatergic and GABAergic neurons in hypothalamus and extended amygdala, also including all non-pallium glutamatergic neurons in cerebral nuclei (1404 clusters)
**MB-HB-CB-GABA**	GABAergic neurons in midbrain, hindbrain, and cerebellum (1040 clusters)
**MB-HB-Glut-Sero-Dopa**	Glutamatergic, serotonergic and dopaminergic neurons in midbrain and hindbrain (1431 clusters)
**NN-IMN-GC**	Non-neuronal cells, immature neurons, granule cells in dentate gyrus, olfactory bulb and cerebellum, and neurons in pineal gland (248 clusters)
**Pallium-Glut**	Glutamatergic neurons from cerebral cortex (derived from the developmental pallium), including isocortex, hippocampal formation, olfactory areas, and cortical subplate (517 clusters)
**Subpallium-GABA**	Telencephalic GABAergic neurons from cerebral cortex and cerebral nuclei, as well as some from the hypothalamic preoptic area, which are derived from the developmental subpallium (1051 clusters)
**TH-EPI-Glut**	Glutamatergic neurons from thalamus and epithalamus (i.e., medial and lateral habenula) (148 clusters)
**WholeBrain**	All cells in the whole mouse brain (5322 clusters)

Table 1. Neighborhoods descriptions.

Description of cells belonging to each neighborhood group.

Table 2. Region acronyms.

Allen Mouse Brain Common Coordinate Framework (CCF) acronyms.

Htr	Defining Feature
Htr1a	Expressed in an important fraction of Sero neurons of the raphe and some HPF excitatory neurons
Htr1b	Expressed in many inhibitory striatal neurons and Sero neurons
Htr1d	Expressed in the striatum, although at much lower levels
Htr1f	Widely expressed in telencephalic structures, especially the Isocortex, with a peak in frontal olfactory structures
Htr2a	Prevalent in glutamatergic cells of the cortical subplate (CLA and EPd) and the mammillary bodies (TMd, PMd), and hippocampal interneurons
Htr2b	Rarely transcribed and is present in some neurons of the pineal gland
Htr2c	Broadly transcribed, especially in the STR, excitatory neurons of the amygdala (LA, BLA and BMA) and RSPv, OLF neurons and structures in MB, P, MY and CB
Htr3a	Uniquely observed in cortical gabaergic neurons of the 06 CTX-CGE GABA class
Htr3b	Uniquely observed in cortical gabaergic neurons of the 06 CTX-CGE GABA class
Htr4	Transcribed at high levels in the OT, excitatory cells of the hippocampus proper and DG, and Chol neurons of the TH (17 MH-LH Glut)
Htr5a	Transcribed at low levels with only one enriched cluster in the MB
Htr5b	Transcribed only in few cells, specifically in Chol neurons of the TH
Htr6	Does not feature any enriched cluster, some cells in CA3 transcribed this Htr
Htr7	Widely transcribed in subcortical structures, especially in some TH nuclei (PF, PVT, IAD and PT), the mammillary complex (MM and PMd), the lateral septal nucleus (LSv) and the fasciola cinerea of the HPF

Table 3. Htrs defining features.

Table listing the defining feature of each Htr trascription pattern.
